# *FLA14* is required for pollen development and preventing premature pollen germination under high humidity in Arabidopsis

**DOI:** 10.1186/s12870-021-03038-x

**Published:** 2021-06-03

**Authors:** Yingjing Miao, Jiashu Cao, Li Huang, Youjian Yu, Sue Lin

**Affiliations:** 1 Institute of Life Sciences, College of Life and Environmental Science, Wenzhou University, Wenzhou, 325000 China; 2 Laboratory of Cell & Molecular Biology, Institute of Vegetable Science, Zhejiang University, Hangzhou, 310058 China; 3 College of Agriculture and Food Science, Zhejiang A & F University, Lin’an, 311300 China; 4 Biomedical Collaborative Innovation Center of Zhejiang Province, Wenzhou, 325000 China

**Keywords:** Arabidopsis, Fasciclin-like arabinogalactan proteins, Microspore development, Intine formation, Pollen germination, High humidity

## Abstract

**Background:**

As an important subfamily of arabinogalactan proteins (AGPs), fasciclin-like AGPs (FLAs) contribute to various aspects of growth, development and adaptation, yet their function remains largely elusive. Despite the diversity of FLAs, only two members, Arabidopsis *FLA3* and rice *MTR1*, are reported to be involved in sexual reproduction. In this study, another Arabidopsis FLA-encoding gene, *FLA14*, was identified, and its role was investigated.

**Results:**

Arabidopsis *FLA14* was found to be a pollen grain-specific gene. Expression results from fusion with green fluorescent protein showed that FLA14 was localized along the cell membrane and in Hechtian strands. A loss-of-function mutant of *FLA14* showed no discernible defects during male gametogenesis, but precocious pollen germination occurred inside the mature anthers under high moisture conditions. Overexpression of *FLA14* caused 39.2% abnormal pollen grains with a shrunken and withered appearance, leading to largely reduced fertility with short mature siliques and lower seed set. Cytological and ultramicroscopic observation showed that ectopic expression of *FLA14* caused disruption at the uninucleate stage, resulting in either collapsed pollen with absent intine or pollen of normal appearance but with a thickened intine.

**Conclusions:**

Taken together, our data suggest a role for *FLA14* in pollen development and preventing premature pollen germination inside the anthers under high relative humidity in Arabidopsis.

**Supplementary Information:**

The online version contains supplementary material available at 10.1186/s12870-021-03038-x.

## Background

In flowering plants, pollen acts as a biological protector of male sperm and is responsible for delivering twin sperm cells via the pollen tube to the embryo sac for double fertilization [1]. Angiosperm pollen ontogenesis is composed of two sequential phases: a developmental phase leading to the formation of mature pollen grains and a functional phase [2]. Male gametophyte development occurs within the anther locules of the stamen. It is a complex process that is initiated by microsporocytes that undergo meiosis to thereby form tetrads of haploid microspores, and it ends with the release of mature pollen grains at the anthesis stage [2, 3]. After the tetrad stage, the precise construction of a multilayered pollen wall controlled by the microspore itself and the sporophytic tapetum is a major event in pollen development [4, 5]. The fundamental structure of the pollen wall is highly similar across species and comprises an inner pectocellulosic intine layer and an outer sporopollenin-based exine layer filled with tryphine (pollen coat) [5]. Exine, whose major component is sporopollenin, is composed of inner nexine and outer sexine that form a three-dimensional structure composed of baculae and a roof-like tectum [6, 7]. The intine layer is the last layer of the pollen wall, consisting of hydrolytic enzymes, hydrophobic proteins, cellulose, hemicellulose, and pectic polymers [5]. Successful male gametophyte development requires coordinated activities of different gametophytic and sporophytic cells and involves widespread appropriate expression of specific genes [8]. The functional phase of pollen ontogenesis begins with the activation of pollen grains by rehydration on the stigma surface, followed by germination and explosive pollen tube growth, and ends with the accomplishment of double fertilization [9]. In Arabidopsis, the progression pattern of proliferating microspores to terminally differentiated pollen is quite clear [10], and tremendous efforts involving genetic and transcriptomic approaches have led to the isolation of several key components related to this unique process, helping to unravel the regulatory mechanism of male gametogenesis, pollen germination, and pollen tube growth [1, 2, 5, 11, 12].

Arabinogalactan proteins (AGPs) are highly glycosylated glycoproteins, that each comprise an O-glycosylated core protein backbone and arabinogalactan polysaccharide chains mainly consisting of galactan and arabinose [13, 14]. AGPs are ubiquitous in all plant tissues and cells and are particularly abundant in the cell wall, plasma membrane, apoplastic space and extracellular matrix [15]. The complexity arising from the incredible diversity of glycans decorating the protein backbone makes AGPs a large complex family in higher plants. To date, a total of 85 and 282 putative AGPs have been identified in the Arabidopsis genome and the *Oryza sativa* genome, respectively [16, 17]. Previous studies have demonstrated that AGPs are extensively implicated in diverse plant growth and development processes under different conditions, such as sexual reproduction (microspore development, female gametogenesis, embryo development, pollen-pistil interaction, pollen germination, and pollen tube growth), vegetative growth (stem development, vascular tissue function, and root development), programmed cell death, cell division and expansion, and response to abiotic stress [13, 15, 18–27].

AGPs are divided into seven subfamilies based on differences in the domain constituents of their core protein backbone sequences: classical AGPs, lysine-rich AGPs, AG peptides, fasciclin-like AGPs (FLAs), nodulin-like AGPs, xylogen-like AGPs, and nonclassical AGPs [13, 22, 28]. FLAs are distinguished from other AGP subfamilies because they contain one or two fasciclin domains (the FAS domain), which may function in cell communication and adhesion (physical interaction) [29–31]. Specifically, the FAS domains are composed of 110–150 amino acids and have low sequence similarity, they contain a conserved central YH motif and two highly conserved regions (H1 and H2), and each region comprises 10 amino acids [30, 32, 33]. Many different putative FLAs have been identified throughout higher plants. For example, 21 FLAs have been identified in Arabidopsis, 27 in *O. sativa*, and 34 in *Triticum aestivum* [30, 31, 34]. Arabidopsis FLAs are divided into four groups (A-D) based on the number and location of FAS and AGP-like domains and the presence or absence of glycosyl phosphatidylinositol (GPI) anchor signals [30]. However, functional investigation of FLAs has been challenged by the redundancy of members, resulting in many *fla* mutants having no discernible phenotypes. At present, the effects of FLAs have been associated with the formation of plant secondary cell walls and wood, shoot and root development, cell adhesion, and responses to abiotic stresses [32]. In addition, the study of *FLA3* in Arabidopsis and *MTR1* in rice revealed the involvement of FLAs in plant reproductive development, especially in pollen formation [35, 36]. However, despite the diversity of FLAs, knowledge of the biological functions of FLAs and of their molecular mechanisms in plants remains elusive.

In this study, a pollen-specific FLA gene, *FLA14*, was isolated and characterized. The loss-of-function mutant of *FLA14* showed no discernible phenotype under standard growth conditions, but premature ectopic pollen germination occurred inside the anthers under high moisture conditions, suggesting an essential role of *FLA14* in maintaining the proper timing of pollen germination. *FLA14*-overexpressing transgenic plants were found to produce approximately 39.2% collapsed and shrunken pollen grains, which displayed decreased pollen germination and pollen tube growth. Cytological observations and electron microscopy revealed that pollen abortion occurred at the uninucleate stage, and aberrant pollen grains lost all cytoplasmic materials, nuclei, and intine, with abnormal distribution of cellulose-like polysaccharides seen by calcofluor white staining, whereas the basic structure of the exine layer and tryphine was normal. These results strongly indicated that ectopic expression of *FLA14* may affect microspore development, especially pollen intine formation.

## Results

### Characterization of *FLA14*

All 21 FLA protein sequences were obtained from the Arabidopsis Information Resource (TAIR) [30]. Using aligned full-length FLA sequences, an unrooted phylogenetic tree showing their phylogenetic relationships was constructed (Additional file 1: Fig. S1a). Based on phylogenetic analysis and pairwise sequence comparison, *FLA14* (AT3G12660), which was identified in the annotated Arabidopsis genome, belongs to group C of the FLA subfamily (Additional file 1: Fig. S1a), which is consistent with previous results [30]. According to the gene expression data obtained from the Arabidopsis eFP Browser and previous transcriptome data of male gametophytes and seedlings in Arabidopsis [37, 38], *FLA14* is a pollen-specific gene that is especially highly expressed in immature tricellular pollen (Additional file 1: Fig. S1b-d). It has a 768-bp-long open reading frame (ORF) with no intron, encoding a 255-amino-acid protein that is rich in Pro (5.9%), Ala (9.0%), Ser (14.5%) and Thr (6.3%) (Additional file 2: Fig. S2a). FLA14 contains only one FAS domain (117 amino acids long) as a key distinguishing feature and has two relatively conserved regions (H1 and H2) (10 and 11 amino acids long, respectively) at either end of the domain (Additional file 2: Fig. S2). In addition, this protein includes an N-terminal secretion signal peptide with a cleavage site between the amino acids at positions 21 (Ser) and 22 (Asn) predicted by SignalP 4.0 Server, TMHMM Server v. 2.0 and SMART, and a potential GPI modification site at amino acid position 225 (Ser) predicted by GPI-SOM and BIG-PI Plant Predictor (Additional file 2: Fig. S2b).

### *FLA14* is specifically expressed in pollen grains

**Fig. 1:**
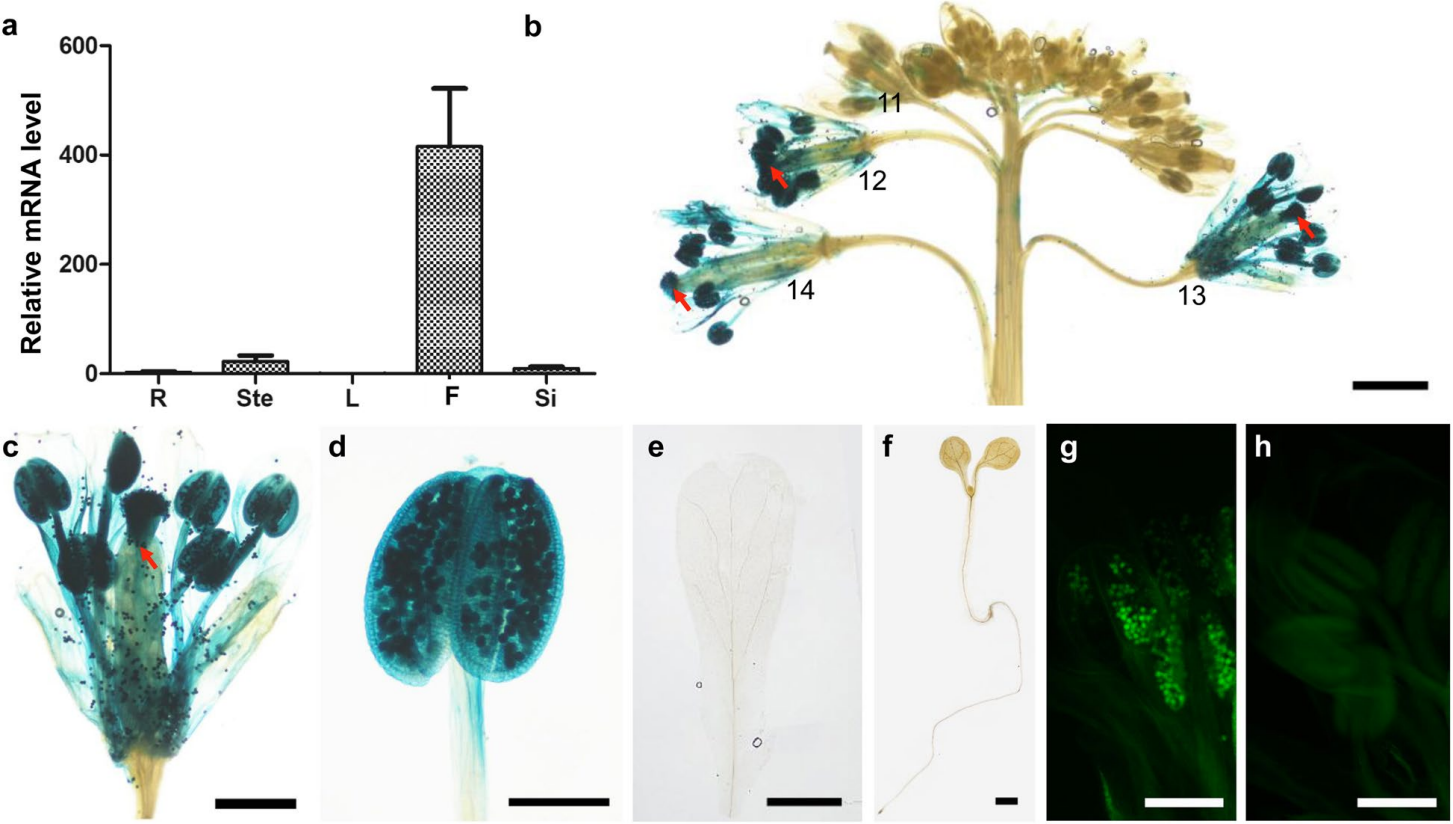
Analysis of *FLA14* expression in Arabidopsis. **a** Quantitative real-time PCR analysis of *FLA14*. The values are the mean ± SD (standard deviation). **b-f** GUS staining in *proFLA14::eGFP-GUS* transgenic plants. **b** A stained inflorescence. Staining was restricted to anthers at floral stages 11–14 and stigmas and styles at floral stages 12–14 (indicated by arrows). **c** Magnified image of a floret at stage 13. The stained transmitting tract is indicated by an arrow. **d** Magnified image of an anther at stage 12. **e** Magnified image of an unstained petal at stage 12. **f** An unstained 9-day-old seedling. **g, h** GFP expression in *proFLA14::eGFP-GUS* pollen grains. **g** Stamens of *proFLA14::eGFP-GUS* flowers compared with wild-type flowers (**h**) at floral stage 12. R, root; Ste, stem; L, young leaf; F, flower; Si, germinal silique. Scale bars = 1 mm (**b**); 500 μm (**c, e, f**); 250 μm (**d, g, h**)

To follow the temporal and spatial expression of *FLA14*, quantitative real-time PCR was first performed in different tissues. The results showed that *FLA14* expression was specifically abundant in flowers but barely detectable in mature roots, stems, leaves or germinal siliques (Fig. 1a). Arabidopsis expressing the *GUS* reporter gene and *eGFP* gene driven by the *FLA14* promoter (*proFLA14::eGFP-GUS*) were constructed and analyzed. According to the stage definition described by Smyth et al. [39], the GUS signal was first detected in anthers at floral stage 11 and rapidly reached a high level in intact anther tissue at floral stage 12 (Fig. 1b). The expanded blue signal in pollen sacs and other floral organs could be the result of the reaction products of GUS enzyme activity diffusing from pollen grains, as the eGFP fluorescent signal in the anthers was only confined to pollen grains, as petals were colorless when stained separately (Fig. 1e, g, h). At floral stage 12 and the anthesis stage, the GUS signal was not only seen in pollen grains but also obvious in the stigma and transmitting tract (Fig. 1b-d). GUS activity was not detected in any other nonreproductive tissues (Fig. 1e, f), which was consistent with the quantitative real-time PCR results. The specific expression pattern of *FLA14* in pollen grains strongly suggested its potential involvement in pollen development and function.

### FLA14 is localized along the plasma membrane and in Hechtian strands

**Fig. 2:**
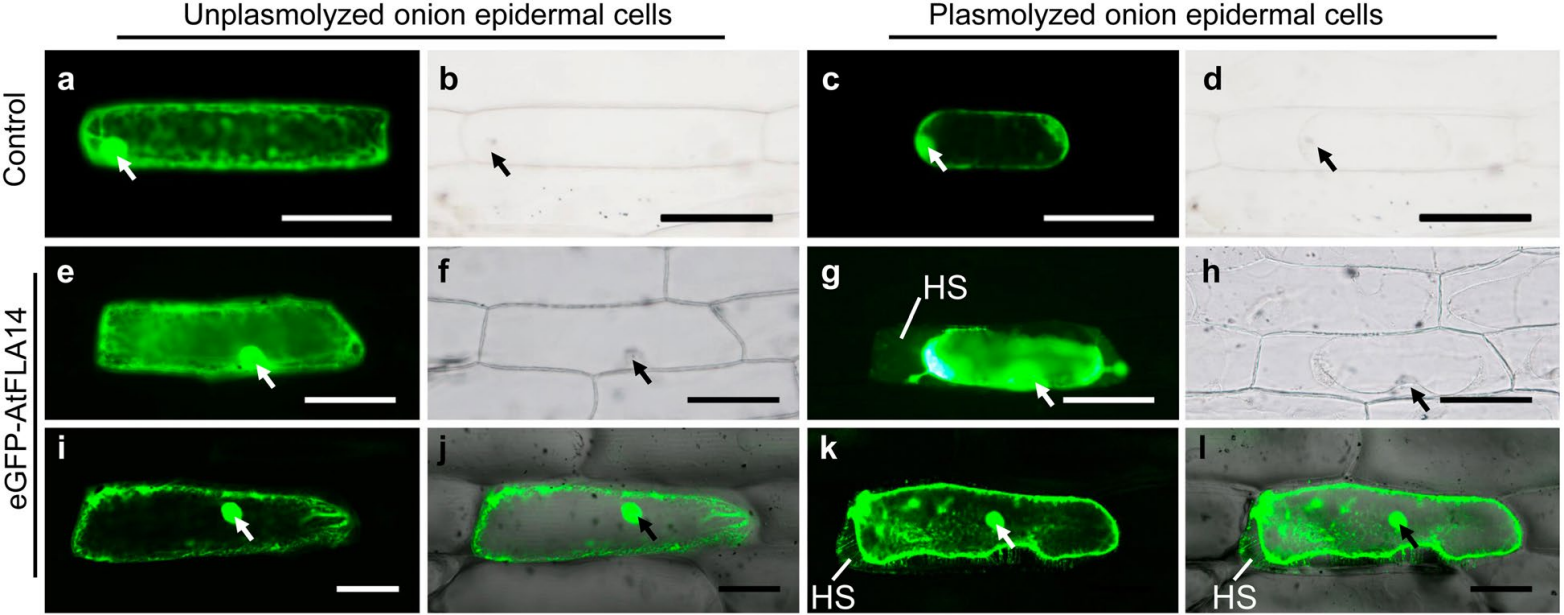
Subcellular localization of the eGFP-FLA14 fusion protein in onion epidermal cells. **a-d** pFGC-eGFP transgenic onion epidermal cells (Control). **e-l** eGFP-FLA14 transgenic onion epidermal cells. **a-h** Images taken by a fluorescence microscope. **i-l** Images taken by a confocal microscope. **a, c, e, g, i, k** Fluorescence images. **b, d, f, h** Bright-field images. **j, l** Overlay of fluorescence and bright-field images. **a, b, e, f, i, j** Unplasmolyzed cells. **c, d, g, h, k, l** Plasmolyzed cells. Arrows indicate the nucleus. HS, Hechtian strands. Scale bars = 100 μm

To investigate the subcellular localization of FLA14, an *eGFP* gene was fused to *FLA14* to form an *eGFP-FLA14* construct under the control of the constitutive CaMV 35S promoter. To avoid potential disturbance of the signal peptide of the FLA14 backbone, eGFP was inserted into FLA14 at the signal peptide cleavage site between the amino acids at positions 21 (Ser) and 22 (Asn). Afterward, this construct was transiently transformed into onion epidermal cells. Our results showed that the fluorescent signal of FLA14 fused with eGFP appeared as a spotty cytoplasmic pattern and displayed broad distribution along the cell boundaries, which was observed on the plasma membrane and in intercellular spaces and Hechtian strands (a stretched plasma membrane extending from the plasmolyzed protoplast to the cell wall in plants) of plasmolyzed cells (Fig. 2e-l), while the fluorescent signal was restricted inside the cell membrane in control cells transformed with a *pFGC-eGFP* empty vector (Fig. 2a-d). These results indicated that FLA14 is a plasma membrane-bound protein.

### Generation of *FLA14* loss-of-function mutant and overexpressing transgenic plants

**Fig. 3:**
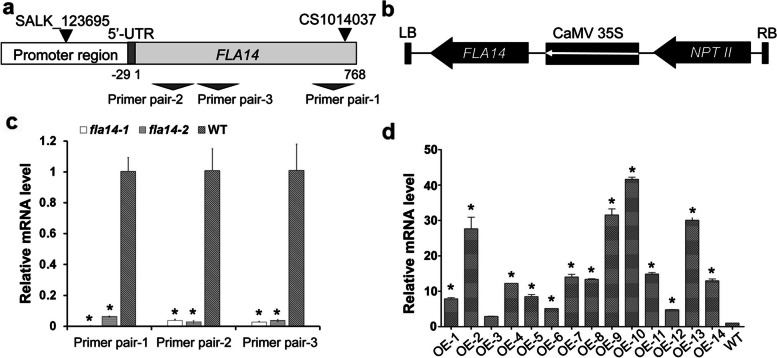
Expression of *FLA14* in Arabidopsis T-DNA-tagged mutant and overexpression (OE) transgenic plants. **a** Schematic representation of the insertion sites for CS1014037 and SALK_123695 mutants. The grayish box represents the coding region, the dark gray box indicates the 5’-untranslated region (5’-UTR) and the white box corresponds to the promoter region. The CS1014037 mutant contains a T-DNA insertion in the coding region of *FLA14* 699 bp downstream of the first ATG. The SALK_123695 mutant contains a T-DNA insertion located 945 bp upstream of the *FLA14* mRNA initiation site. Three dark gray triangles indicate the position of three primer pairs used for quantitative real-time PCR analysis. **b** Map of the T-DNA portion of the binary vector used in the *FLA14* OE construct. **c, d** Quantitative real-time PCR analysis of *FLA14* in the homozygous CS1014037 line (*fla14-1*) and SALK_123695 line (*fla14-2*) (**c**) and the OE lines (**d**) compared with wild-type (WT) plants. The values are the mean ± standard deviation (SD). Asterisks indicate significantly different means (*p* < 0.05) using one-way ANOVA. RNA is extracted from whole inflorescences. Ubiquitously expressed *Tubulin beta-4* was used as an internal control

To study the biological function of *FLA14*, two mutants (CS1014037 and SALK_123695) harboring T-DNA insertions, one in the coding region of *FLA14* 699 bp downstream of the initiation codon (ATG) and the other located 945 bp upstream of the *FLA14* mRNA initiation site, were identified (Fig. 3a). Additionally, we generated *FLA14* overexpression (OE) transgenic plants (Fig. 3b). The homozygous CS1014037 mutant *fla14-1*, homozygous SALK_123695 mutant *fla14-2* and 14 OE-positive transformants were verified by genomic DNA PCR (Additional file 3: Fig. S3). In the quantitative real-time PCR results, weak or almost no *FLA14* mRNA could be detected in the whole inflorescences of *fla14-1* and *fla14-2* (Fig. 3c), with three specific primer pairs (Additional file 4: Table S1 and Fig. 3a), indicating that transcription of the *FLA14* gene was strongly suppressed by T-DNA insertion. Moreover, compared with wild-type (WT) inflorescences, significantly enhanced *FLA14* transcript levels in the inflorescences from 13 OE lines were confirmed (Fig. 3d).

### Mutation in *FLA14* results in precocious pollen germination inside the anthers under high moisture conditions

**Fig. 4:**
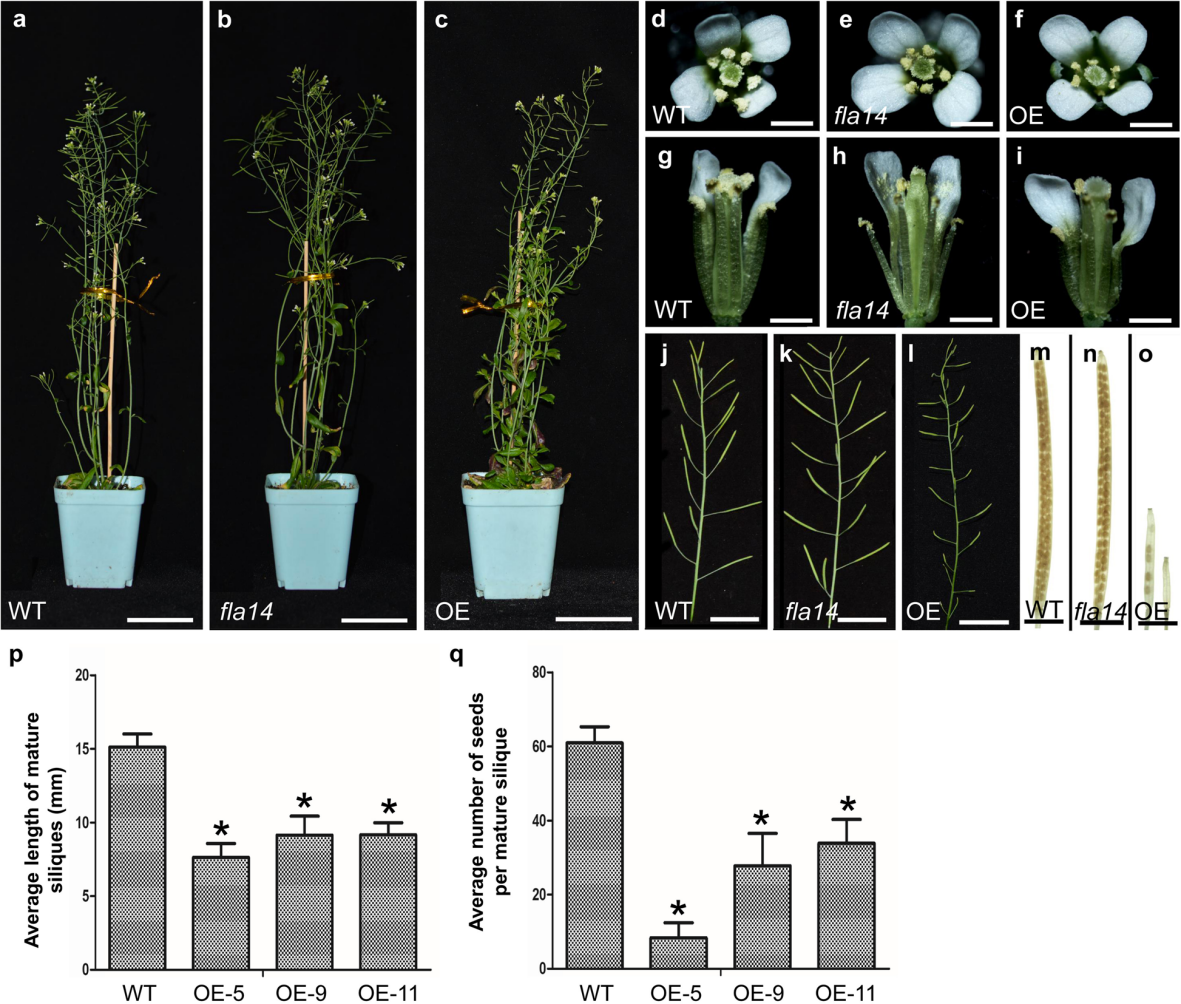
Fertility analysis of *FLA14* knockout mutant (*fla14*) and overexpression (OE) transgenic Arabidopsis plants. **a-c** Morphological observation of wild-type (WT) (**a**), *fla14* (**b**), and OE (**c**) plants. **d-i** A WT floret (**d, g**), a *fla14* floret (**e, h**), and an OE floret (**f, i**). **j-l** Comparison of silique development in WT (**j**), *fla14* (**k**), and OE (**l**) plants. The OE siliques were smaller than the normal siliques of *fla14* and WT plants. **m–o** Ethanol-fixed siliques of WT (**m**), *fla14* (**n**), and OE (**o**) plants. The OE siliques show few mature ovules. **p** Length of mature siliques in WT and OE plants. The values are the mean ± standard deviation (SD). Asterisks indicate significantly different means (*p* < 0.01) using one-way ANOVA. **q** Number of seeds per silique in WT and OE plants. The values are the mean ± standard SD. Asterisks indicate significantly different means (*p* < 0.01) using one-way ANOVA. Scale bars = 5 cm (**a-c**); 1 mm (**d-i**); 2 cm (**j-l**); 5 mm (**m–o**)

**Fig. 5:**
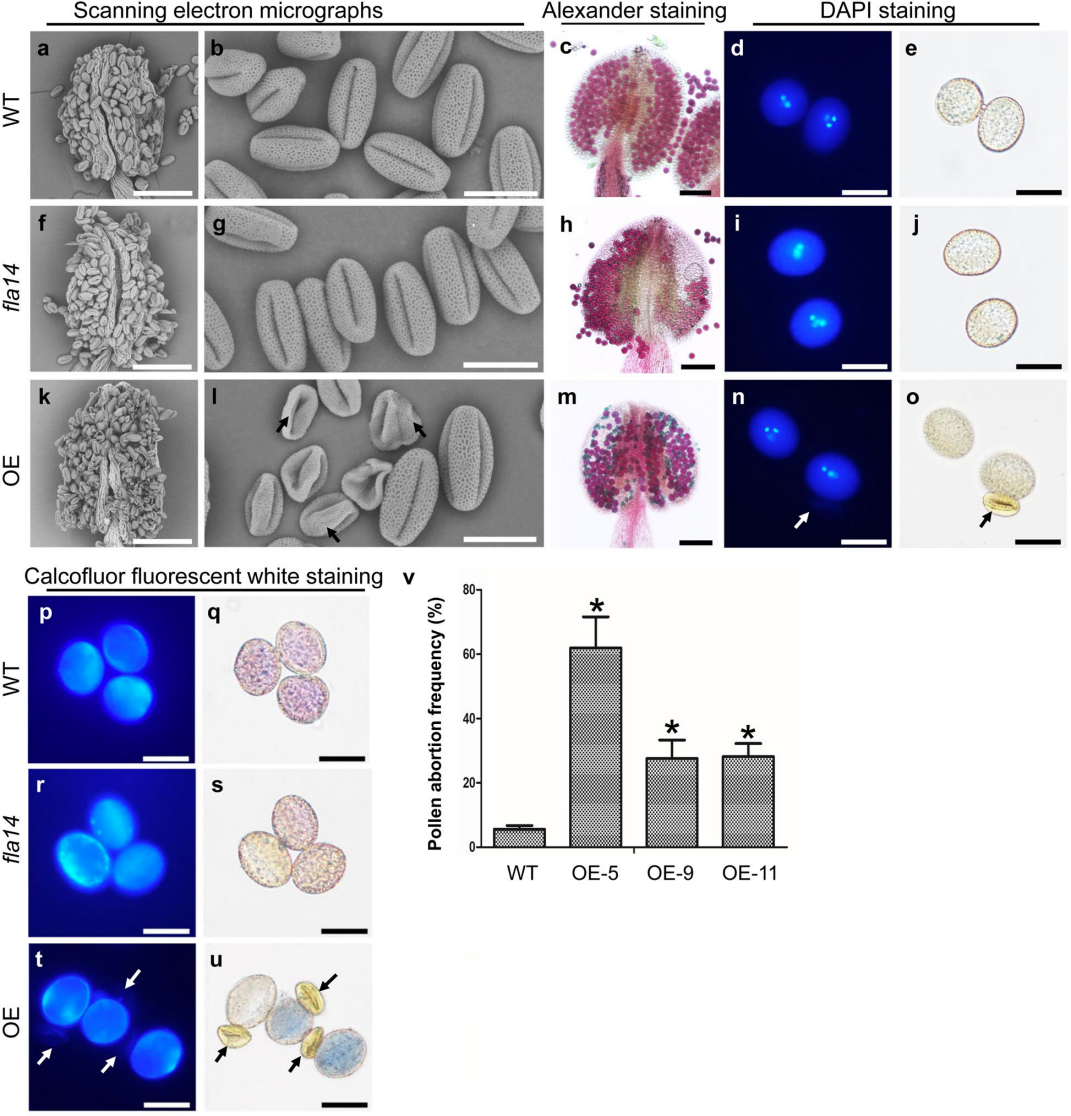
Mature pollen analysis in *FLA14* knockout mutant (*fla14*) and overexpression (OE) transgenic Arabidopsis plants. **a, f, k** Scanning electron micrographs of a wild-type (WT) anther (**a**), a *fla14* anther (**f**), and an OE anther (**k**) at the time of dehiscence. **b, g, l** Scanning electron micrographs of mature pollen grains in WT (**b**), *fla14* (**g**), and OE (**l**) plants. **l** Arrows indicate the aborted pollen grains that have a collapsed appearance. **c, h, m** Alexander staining of mature pollen grains in WT (**c),***fla14* (**h**), and OE (**m**) plants. The unviable pollen grains are dyed greenish black. **d, e, i, j, n, o** 4’,6-diamidino-1-phenylindole (DAPI) staining of mature pollen grains in WT (**d**), *fla14* (**i**), and OE (**n**) plants. **e, j, o** The corresponding bright field images. **n, o** Arrows indicate the aborted pollen grains with no detectable nuclei. **p-u** Calcofluor fluorescent white staining of mature pollen grains in WT (**p**), *fla14* (**r**), and OE (**t**) plants. **q, s, u** The corresponding bright field images. **t, u** Arrows indicate the aborted pollen grains with no detectable calcofluor white fluorescence. **v** Pollen abortion frequencies in WT and OE plants. The values are the mean ± standard SD. Asterisks indicate significantly different means (*p* < 0.01) using one-way ANOVA. Scale bars = 100 μm (**a, c, f, h, k, m)**; 20 μm (**b, d, e, g, i, j, l, n-u**)

The loss-of-function mutants of *FLA14*, *fla14-1* and *fla14-2* displayed consistent plant growth and reproductive development phenotypes. In addition, we observed that under standard growth conditions, there were no obvious morphological differences between *fla14* (Fig. 4b) and WT plants (Fig. 4a). The opening florets of *fla14* had a normal appearance, with yellow and plump anthers (Fig. 4e, h), similar to WT florets (Fig. 4d, g), and mature siliques showed no reduction in length or seed set (Fig. 4k, n). Normal-appearing pollen grains with precisely positioned germinal apertures were observed in the anthers of *fla14* (Fig. 5f, g), which were similar to WT pollen grains (Fig. 5a, b). Cytological observations revealed that *fla14* pollen grains displayed normal pollen viability, nuclear distribution, and deposition of cellulose-like polysaccharides (Fig. 5h-j, r, s). In vivo self and reciprocal crosses between *fla14* and WT plants further confirmed that neither the male nor female gametophytes of *fla14* were impaired (Additional file 5: Fig. S4).

**Fig. 6:**
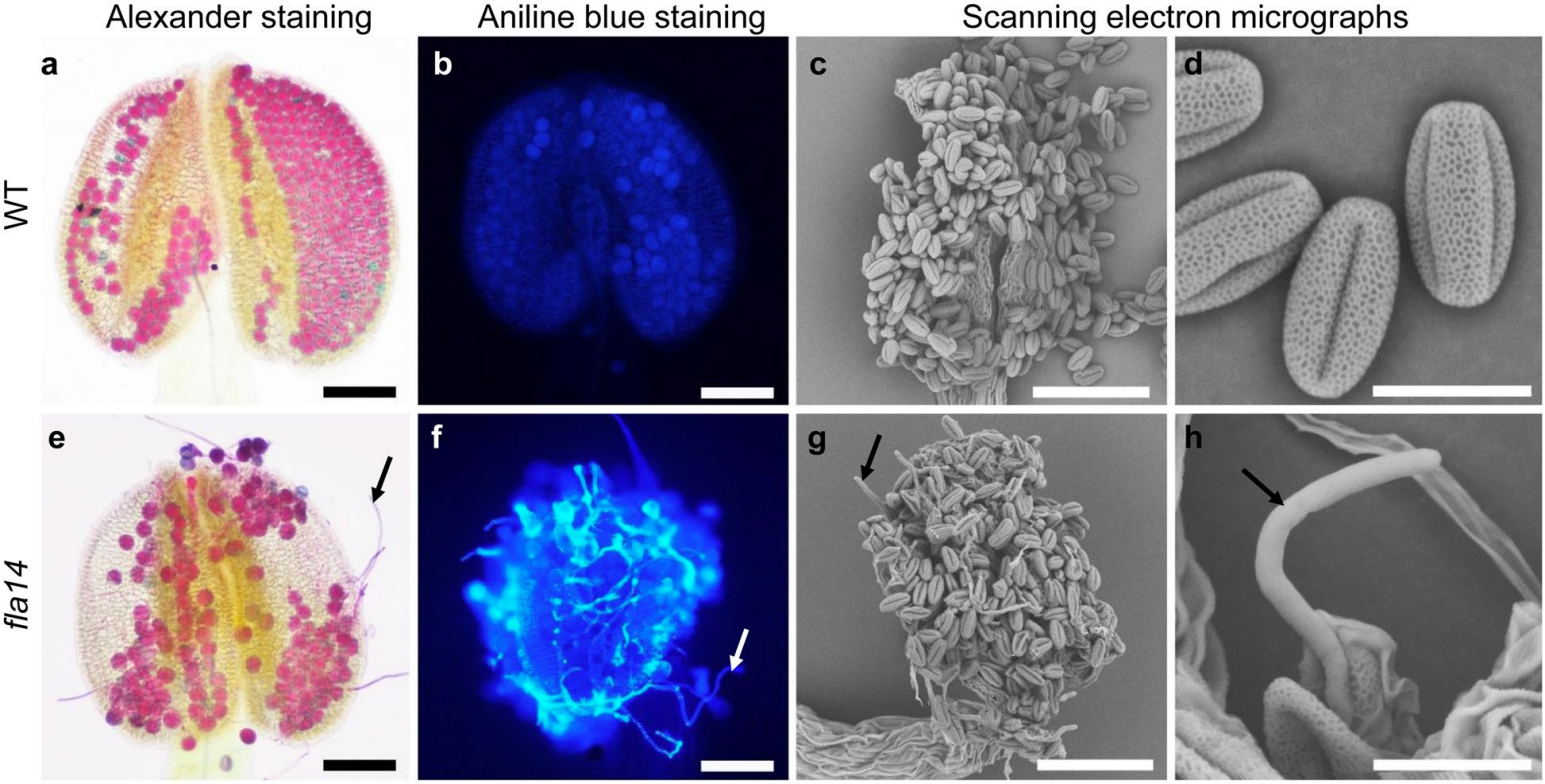
In vivo pollen germination at 85% relative humidity in the *FLA14* knockout mutant (*fla14*) of Arabidopsis. **a, e** Alexander staining of dehiscent anthers from wild-type (WT) (**a**) and *FLA14* knockout mutant (*fla14*) (**e**) plants. **b, f** Fluorescence microscopy images of a WT anther (**b**) and a *fla14* anther (**f**) at the time of dehiscence stained with aniline blue. Pollen tubes germinating and elongating within a *fla14* anther (**f**) are labeled with aniline blue to show callose. **c, g** Scanning electron micrographs of dehiscent anthers from WT (**c**) and *fla14* (**g**) plants. **d, h** Scanning electron micrographs of pollen grains and pollen tubes from WT (**d**) and *fla14* (**h**) plants. **e–h** Arrows indicate apparent pollen tubes germinating and elongating within the anthers of *fla14* plants grown at 85% relative humidity. Scale bars = 100 μm (**a-c, e–g**); 20 μm (**d, h**)

However, it is worth noting that pollen germination and elongation were determined to be altered when *fla14* was transferred to high moisture conditions with a relative air humidity of 85%. In *fla14*, a considerable number of highly elongated pollen tubes were consistently observed inside the anthers at 85% humidity (Fig. 6e-h). In contrast, precocious germination of pollen tubes was never observed in the anthers of WT plants under either controlled (50%) (Fig. 5f-h) or high (85%) (Fig. 6a-d) relative humidity conditions.

### *FLA14* overexpression in transgenic plants causes microspore abortion at the uninucleate stage

OE plants without any apparent vegetative defects showed severe sterility compared with WT plants (Fig. 4j, l, m, o). We randomly selected three OE lines (5, 9, and 11) with markedly enhanced *FLA14* transcript levels for further analysis. The mature siliques of OE plants (8.66 ± 1.26 mm) were obviously shorter than those of WT plants (15.11 ± 0.92 mm) (Fig. 4p), producing much fewer seeds (23.9 ± 13.5 per silique) than WT plants (60.9 ± 4.4 per silique) (Fig. 4q). OE plants displayed a partial male-sterile phenotype with approximately 39.2% distorted pollen grains (Fig. 5k, l, v), while only a 5.5% pollen abortion frequency was observed in WT plants (Fig. 5a, b, v). Cytological observations revealed that these collapsed pollen grains from the opening flowers were unviable and lacked cell content and nuclei, and the deposition of cellulose-like polysaccharides seemed to be aberrant when traced by calcofluor white staining (Fig. 5m-o, t, u).

**Fig. 7:**
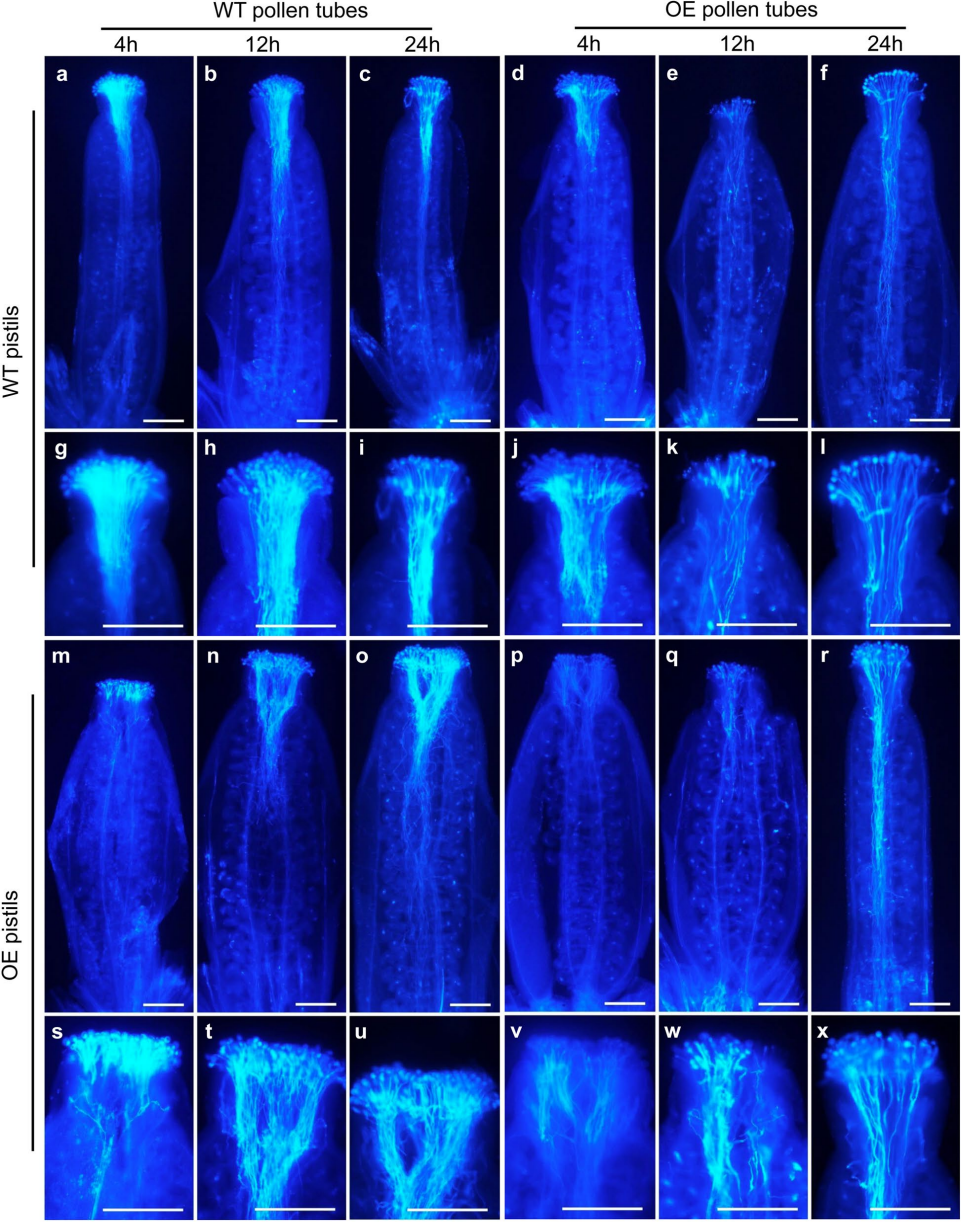
Self and reciprocal crosses between wild-type (WT) and *FLA14*-overexpressing (OE) transgenic Arabidopsis plants. **a-c** WT pollen tubes in WT pistils at 4 h after pollination (HAP), 12 HAP, and 24 HAP, respectively. **d-f** Pollen tubes of OE plants in WT pistils at 4 HAP, 12 HAP, and 24 HAP, respectively. **m–o** WT pollen tubes in OE pistils at 4 HAP, 12 HAP, and 24 HAP, respectively. **p-r** Pollen tubes of OE plants in OE pistils at 4 HAP, 12 HAP, and 24 HAP, respectively. **g-l** and **s-x** The corresponding magnified images of (**a-f**) and (**m-r**). Scale bars = 200 μm

Further analysis of pollen tube behavior via self and reciprocal crosses was also conducted using aniline blue to trace callose. When WT pollen was employed as the male donor, comparison of OE (Fig. 7m-o, s-u) and WT (Fig. 7a-c, g-i) pistils failed to reveal differences in pollen tube behavior. However, when pollen from the OE plants was employed as the male donor, the amount of pollen tubes in OE (Fig. 7p-r, v-x) and WT (Fig. 7d-f, j-l) pistils was obviously lower than that of WT pollen tubes in both OE (Fig. 7m-o, s-u) and WT (Fig. 7a-c, g-i) pistils, although the pollen tube behavior in OE and WT pistils was similar. Therefore, aniline blue staining analysis demonstrated that the male gametophytes of OE plants were impaired.

**Fig. 8:**
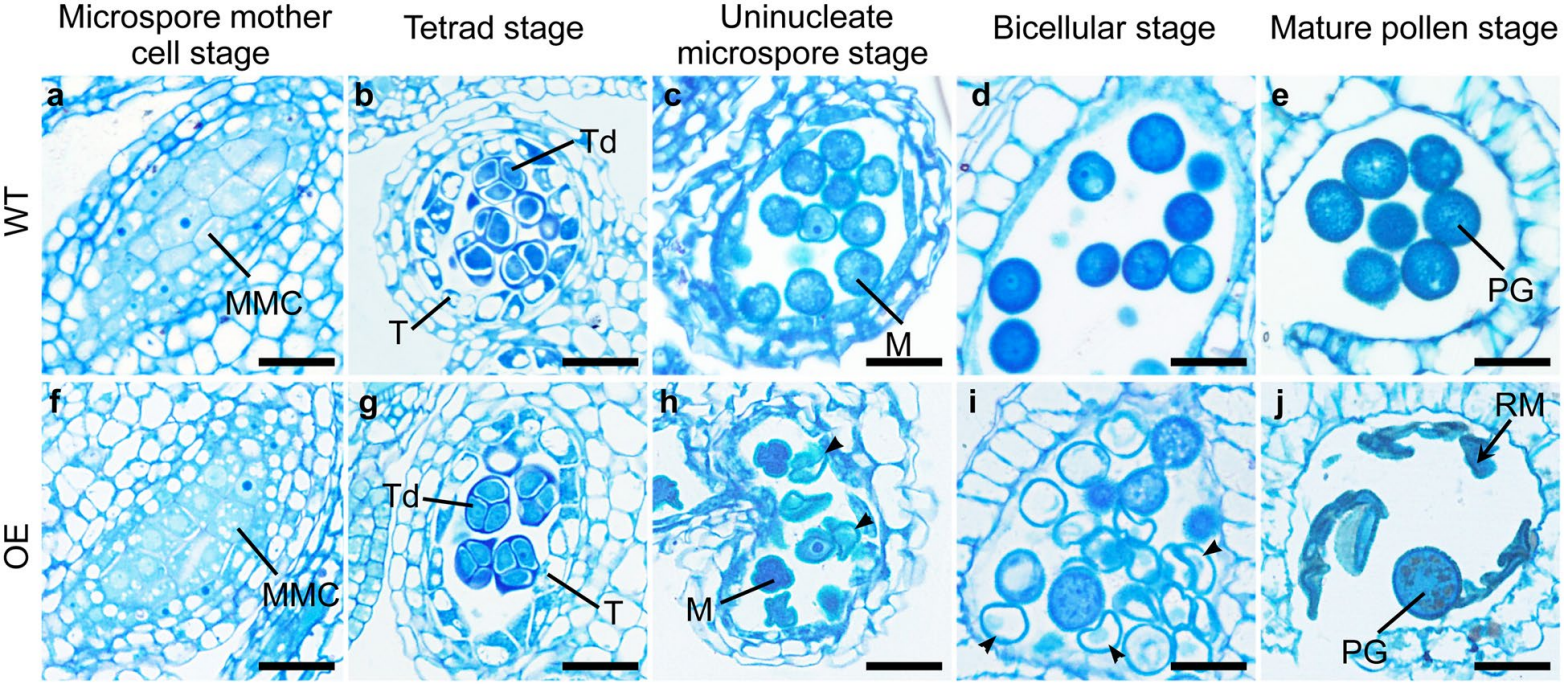
Semithin sections of anthers from wild-type (WT) and *FLA14*-overexpressing (OE) transgenic Arabidopsis plants. **a-e** Anthers from WT plants. **f-j** Anthers from OE plants. **a, f** Microspore mother cell stage. **b, g** Tetrad stage. **c, h** Uninucleate microspore stage. Arrowheads in (**h**) indicate the aborted microspores. **d, i** Bicellular stage. Arrowheads in (**i**) indicate the aborted microspores. **e, j** Mature pollen stage. Arrows in (**j**) show the remnants of the aborted microspores. M, microspore; MMC, microspore mother cell; PG, pollen grain; RM, remnants of microspores; T, tapetum; Td, tetrads. Scale bars = 20 μm

To investigate the precise stage during which pollen collapse started due to the enhanced *FLA14* transcript levels, floral buds from the OE plants were fixed, embedded and sectioned for analysis of anther development. The sporophytic tapetum of OE anthers appeared normal, were fully developed by the tetrad stage, and gradually degenerated during the late stage of the microspore development process (Fig. 8f-j), with no obvious distinction compared with the tapetum of WT anthers (Fig. 8a-e). However, the maturation process of OE pollen grains prior to anther dehiscence was distinguished since the uninucleate stage (Fig. 8h). A total of 27.6–61.9% bicellular microspores of OE plants were accompanied by extensive degeneration of all cytoplasmic contents (Fig. 8i) and formed remnants at the dehiscent stage (Fig. 8j). Therefore, semithin section analysis demonstrated that only the microspore development process was impaired in OE plants, with no defects in tapetum formation and degradation.

**Fig. 9:**
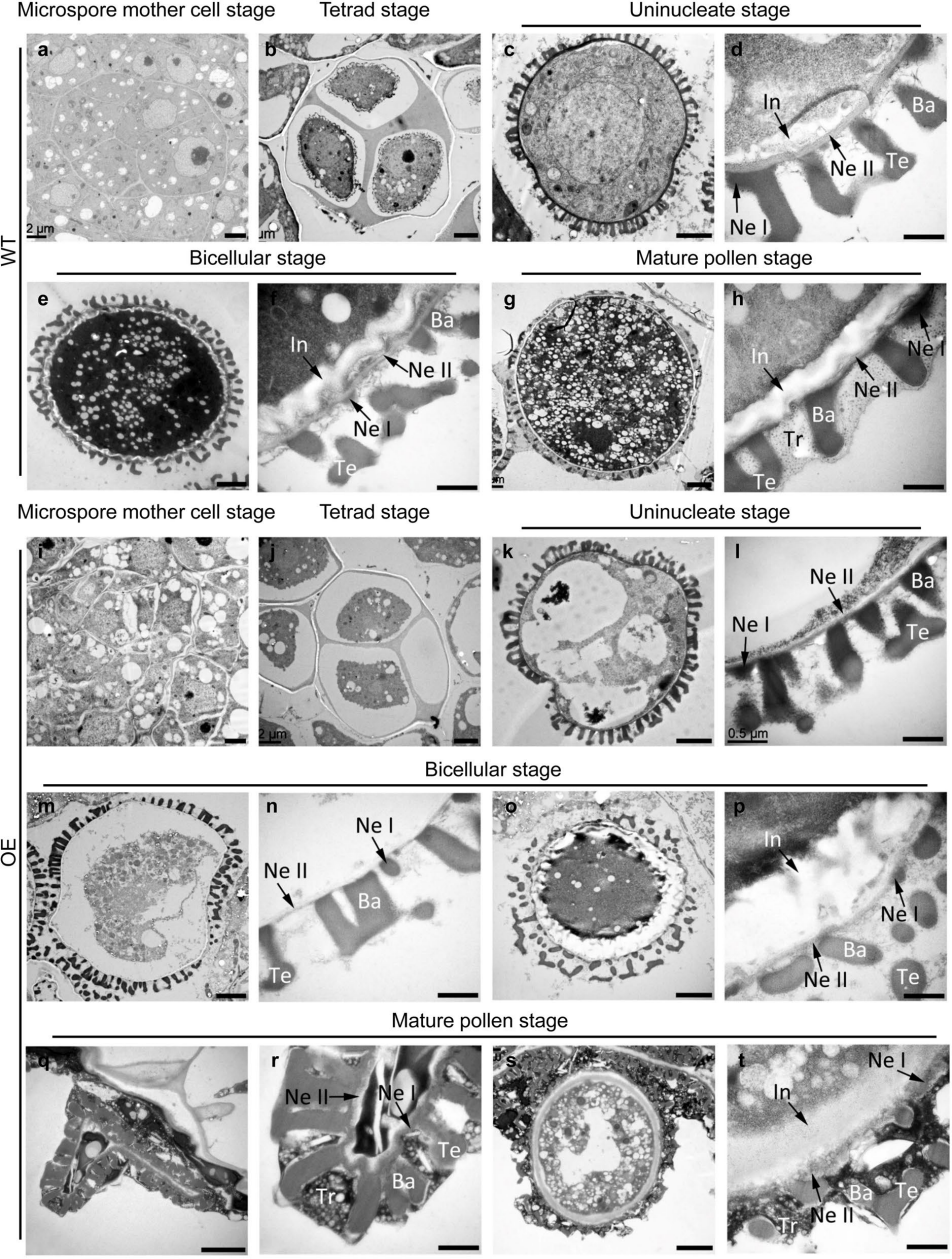
Transmission electron micrographs of microspores from wild-type (WT) and *FLA14*-overexpressing (OE) transgenic Arabidopsis plants. **a-h** Microspores from WT plants. **i-t** Microspores from OE plants. **a, i** Microspore mother cell stage. **b, j** Tetrad stage. **c, d, k, l** Uninucleate microspore stage. The degradation of the cytoplasm is evident in (**k**). **e, f, m-p** Bicellular stage. The degenerated microspore is shown in (**m**). Abnormal pollen wall patterning is visible in (**o**). **g, h, q-t** Mature pollen stage. The mature pollen grain in (**q**) is devoid of any content and nuclei. The pollen wall structure in (**s, t**) is aberrant. **d, f, h, l, n, p, r, t** Detailed structure of pollen walls in (**c, e, g, k, m, o, q, s**). Intine layers are absent in (**l, n, r**). The abnormal thickening of intine layers in (**p, t**) is obvious. Ba, baculum; Ex, exine; In, intine; Ne I, nexine I; Ne II, nexine II; Te, tectum; Tr, tryphine. Scale bars = 20 μm (**a-c, e, g, i-k, m, o, q, s**); 0.5 μm (**d, f, h, l, n, p, r, t**)

Abnormalities of OE microspores and their pollen wall structure, which was gradually constructed during pollen development, were further confirmed by TEM. Most aberrant pollen grains in OE plants showed a collapsed appearance (Fig. 9q), in contrast with the roughly round pollen grains of WT plants (Fig. 9g). From the microspore mother cell stage to the tetrad stage, all the developing cells in the OE anther locules (Fig. 9i, j) exhibited the same morphology and pollen wall patterning as those of WT plants (Fig. 9a, b). The young microspores surrounded by the callose wall at the tetrad stage showed the initiation of exine wall formation. However, degradation of the cytoplasm was evident at the uninucleate stage (Fig. 9k), which was different from observations of individual haploid WT microspores (Fig. 9c). Moreover, the intine layer of OE microspores was unable to form at this point (Fig. 9l) compared with the presence of almost complete basic intine and exine structures in WT microspores (Fig. 9d). Subsequently, the degradation of the cytoplasmic content and the failure of intine formation led to the fading of nuclei and the collapse of bicellular microspores (Fig. 9m). By the dehiscent stage, the OE anthers released empty and shrunken pollen grains that were completely devoid of cell contents and an intine layer but with an intact exine layer and a well-developed tryphine (Fig. 9q, r). In addition, some sporadic cases of aberrant pollen grains with a normal appearance but remarkable thickening of the intine layer were observed inside the OE pollen sac (Fig. 9o, s). This kind of pollen grain underwent similar development to WT pollen grains before the uninucleate stage. However, dramatic overdeposition of the intine layer became apparent starting from the bicellular stage (Fig. 9p, t). All these results indicated that microspore abortion in OE plants occurred at the uninucleate stage and suggested a role of *FLA14* in microspore development and pollen wall patterning in Arabidopsis, especially in intine formation.

### The deposition of cellulose-like polysaccharides during pollen wall formation is impaired in *FLA14*-overexpressing transgenic plants

**Fig. 10:**
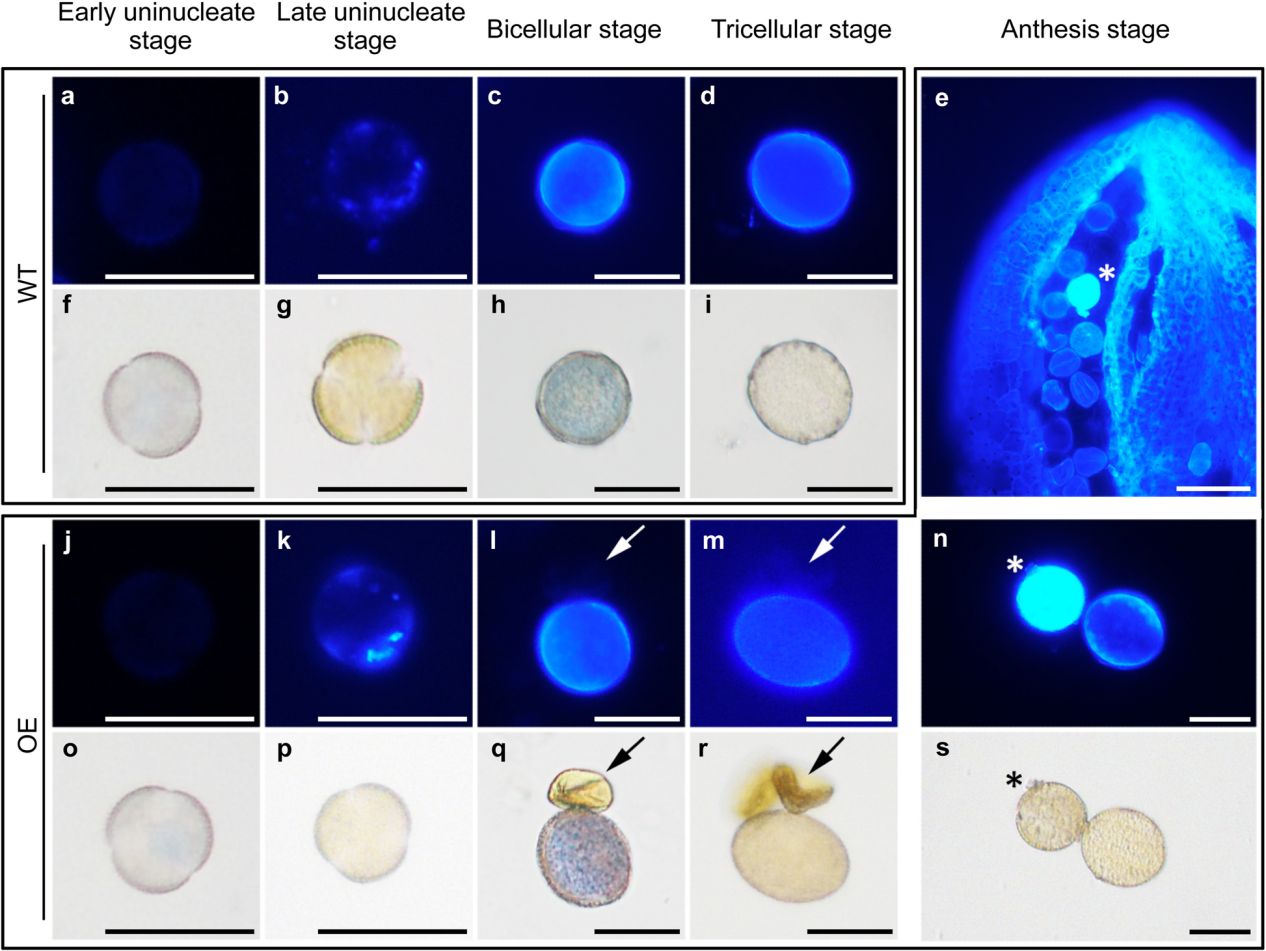
Calcofluor white staining of microspores from wild-type (WT) and *FLA14*-overexpressing (OE) transgenic Arabidopsis plants. **a-d, f-i** Microspores from WT plants. **f-i** The corresponding bright field images. **e** An anther at anthesis stage from OE plants. Asterisks indicate aberrant pollen grains with a significant increase in the calcofluor white fluorescent signals. **j-s** Microspores from OE plants. **o-s** The corresponding bright field images. **l, m, q, r** Arrows indicate the aborted pollen grains with no detectable calcofluor white fluorescence. **n, s** Comparison of aberrant pollen grains with unusually strong calcofluor white fluorescence (indicated by asterisks) and normal mature pollen grains from OE plants. **a, f, j, o** Early uninucleate stage. **b, g, k, p** Late uninucleate stage. **c, h, l, q** Bicellular stage. **d, i, m, r** Tricellular stage. **e, n, s** Anthesis stage. Scale bars = 20 μm (**a-d, f-s**); 50 μm (**e**)

To determine how *FLA14* affects intine formation, we used calcofluor white staining to trace the deposition of cellulose-like polysaccharides during the formation of the pollen wall in OE plants. Very faint calcofluor white fluorescence was first detected in OE (Fig. 10j, o) and WT microspores (Fig. 10a, f) at the early uninucleate stage and rapidly formed obvious fluorescent spots at the late uninucleate stage (Fig. 10b, g, k, p). In WT microspores, the intine displayed a distinct fluorescent ring outside the cytoplasm at the bicellular and tricellular stages (Fig. 10c, d, h, i). However, some small and aborted pollen grains with no detectable calcofluor white fluorescent signals were found in OE plants at the bicellular and tricellular stages (Fig. 10l, m, q, r). In addition, pollen grains that were normal in shape but that had enhanced calcofluor white fluorescence signals were also observed at a very low proportion in OE plants (Fig. 10e, n, s). This phenomenon of unusual enhancement of fluorescence signals has never been observed in WT pollen grains. The correlation between the abnormal distribution of calcofluor white fluorescence (no fluorescence or a significantly enhanced fluorescent signal) and the aberrant intine patterning (failed to form or markedly thickened) reflected in TEM (Fig. 9) suggested that ectopic expression of *FLA14* may affect Arabidopsis pollen intine formation probably through disturbing the deposition of cellulose-like polysaccharides.

## Discussion

### Ectopic expression of *FLA14* leads to impaired microspores with defective pollen intine formation

In recent years, a set of investigations have identified a batch of AGPs with specific expression and function in male reproductive tissues, demonstrating the undoubtedly important role of AGPs in pollen ontogenesis [20, 34, 35, 40–42]. However, to our knowledge, there are currently only two FLAs, Arabidopsis *FLA3* and rice *MTR1*, that are reported to be endowed with a specific role in microspore development [35, 36]. In this study, we identified a FLA-encoding gene, *FLA14*, which displayed a strict pollen-specific expression pattern (Fig. 1). To study the function of *FLA14*, mutants harboring T-DNA insertions and CaMV 35S promoter-driven *FLA14*-OE transgenic Arabidopsis plants were generated. The activity of the CaMV 35S promoter in Arabidopsis pollen has always been controversial. However, the CaMV 35S promoter is still widely used in the functional analysis of pollen-expressed genes. Taking AGP family members as an example, CaMV 35S promoter-driven RNAi plants in Arabidopsis were generated to study the function of *AGP6* and *AGP11*, both of which were found to be specifically expressed in stamens, pollen grains and pollen tubes [41, 43]. Downregulation of *FLA3*, which is specifically expressed in pollen grains and tubes, caused pollen abnormalities in CaMV 35S promoter-driven RNAi plants [35]. Overexpression of *FLA3* under the CaMV 35S promoter also led to marked sterility [35]. Additionally, transgenic plants of *Brassica campestris* expressing antisense *BcMF8* and/or *BcMF18*, which are specifically expressed in pollen grains and/or pollen tubes, under the control of the CaMV 35S promoter showed partial male sterility and decreased seed set [42, 44]. In our study, analysis of *fla14* mutants and *FLA14*-overexpressing transgenic Arabidopsis plants controlled by the CaMV 35S promoter was performed.

The data presented here indicated that membrane-bound FLA14 is associated with the proper progression of pollen development (Fig. 2, 5). Although early results from *fla14* null-mutant characterization showed no alteration in plant phenotype under standard growth conditions (Fig. 4, 5), the effects of *FLA14* overexpression were evident as early as the uninucleate stage during the microspore development process (Fig. 8, 9). Notably, the initiation and timing of exine formation in OE plants was normal and similar to that in WT plants (Fig. 9). However, unlike in WT plants, intine in OE plants did not form properly and was followed by the degeneration of microspore cytoplasm. Moreover, in accordance with the intine defects, our calcofluor white staining results showed an absence or excess of fluorescence signals in the aborted pollen grains of OE plants, which demonstrated abnormal distribution of cellulose-like polysaccharides in the pollen grains (Fig. 10). All these results suggested that ectopic expression of *FLA14* is likely to disturb the distribution of cellulose-like polysaccharides in developing pollen cells.

Thus, the question arises of how ectopic expression of *FLA14* affects the distribution of cellulose-like polysaccharides in OE pollen. As the innermost layer surrounding the microspores, intine is composed of multiple components (cellulose, hemicellulose, pectin, hydrolytic enzymes and structural proteins) that are covalently and noncovalently linked [45]. Protein glycosylation is a cotranslational or posttranslational covalent attachment of carbohydrate chains to the polypeptide backbone, which is an essential modification for proper protein structure and function in the male gametophyte [46]. Proteoglycans are important components of the pollen wall, among which AGPs are a prominent family of highly glycosylated glycoproteins [13]. Nevertheless, the assembly of AGPs within the pollen wall system and the connection with other extracellular matrix components are poorly understood. Previous studies have demonstrated that AGPs may serve as covalent cross-linkers and plasticizers in the cell wall and form linkages with other components of the cell wall, mainly pectin and arabinoxylan [47, 48]. This continuous proteoglycan structure network in consequently influences extracellular matrix integrity and helps to maintain cell expansion [48, 49]. For instance, an Arabidopsis AGP (AGP57C) was proposed to covalently interact with pectins and hemicelluloses. In addition, *BcMF18* in *B. campestris* was suggested to be an integral part of pollen intine formation, possibly as a cross-linker in the formation of proteoglycan structure [50]. Hence, we hypothesize that periplasm-AGPs are good candidates for the proteoglycan structure in pollen intine and may help to maintain intine integrity, providing a safe interior environment for microspore development. Therefore, overexpression of *FLA14* disorders the homeostatic level of proteoglycan during microspore expansion, resulting in changes in structural components and abnormal intine construction (Fig. 9). Subsequently, abnormal intine construction further affects the pollen internal environment and leads to the degradation of the cytoplasmic content in OE pollen grains (Fig. 9), as the intine layer is considered to play an important role in pollen stability and integrity [35, 50, 51].

### *FLA14* is essential for preventing premature pollen germination inside anthers under high relative humidity in Arabidopsis

The presence of AGPs within pollen tubes has been documented in diverse plant species, mainly at their apex [27, 52, 53]. A specific subset of AGPs was shown to be not only important for pollen development but also good candidates to mediate pollen tube growth. In addition, some specific members of AGPs were reported to be essential for preventing premature pollen grain germination. AGP6 and AGP11 are a pair of redundant AGPs involved in Arabidopsis pollen development and are necessary for proper pollen tube growth as well as for preventing premature germination of pollen grains inside anthers under high relative humidity [54]. AGP40 is an AG peptide that shows a high expression level in pollen grains and pollen tubes. Although AG peptides are very different molecules, typically consisting of fewer than 30 amino acid residues, AGP40 presents a high degree of similarity with and is closely related to AGP6 and AGP11. The single null mutant of *AGP40* lacks obvious phenotypic differences when compared with WT plants however, a significantly higher number of early germinating pollen tubes inside the anthers were observed in *agp6 agp11 agp40* triple mutants [20]. In our study, the results of preliminary characterization of the knockout mutant *fla14* showed no alteration in microspore development, whereas high humidity affected the *fla14* phenotype, resulting in a considerable number of precociously germinated pollen grains with over elongated pollen tubes inside mature anthers (Fig. 6). Precocious pollen germination was never observed in WT plants, even under conditions of high relative humidity. It is suggested that *FLA14* is responsible for preventing premature pollen germination inside the anthers.

In previous studies, several mutants and overexpression transgenic plants with precocious pollen germination in anthers have been reported in Arabidopsis. The first known mutant with this novel defect, *raring-to-go*, showed precociously germinating pollen inside anthers in a humidity-dependent manner; unfortunately, the gene harboring this mutation has not been identified to date [55]. Arabidopsis *plantacyanin* overexpression resulted in defects in anther dehiscence, leading to a low level of precociously germinated pollen grains inside the closed anthers. A hypothesis was proposed that pollen may interpret the presence of plantacyanin in mature anthers as a stigmatic signal and hence respond by germinating [56]. In addition, a loss-of-function mutant *callose synthase 9* and a transgenic plant overexpressing *Callose Synthase 5* both contain abnormal callose deposition during microsporogenesis and show precocious pollen germination [57]. Studies of these well-characterized mutants or overexpression plants in Arabidopsis demonstrate that precocious pollen germination could be triggered by a genetic alteration. However, unlike pollen grains from these mutants or overexpression lines, which do germinate under normal culture conditions, the premature germination of pollen grains in the anthers from *fla14* and *agp6 agp11* is dependent on high humidity.

Normally, pollen tube germination and directional growth occur after anthesis, and premature germination inside anthers does not occur [54]. To date, dehydration is considered to be the main factor in maintaining pollen dormancy and preventing precocious germination [58]. Pollen adhesion and rehydration break dormancy and are postulated to be the initial steps for triggering pollen germination [59]. However, the underlying mechanisms of these events are still poorly understood. It has been reported that mutations in *OsOSC12*/*OsPTS1*, encoding a bicyclic triterpene synthase, resulted in defective pollen adhesion and hydration in rice under low-humidity conditions [60]. High humidity can rescue the defective pollen hydration resulting from loss-of-function mutants of *OsGL1-4*, a member of the Glossy family in rice [61]. Altered environmental humidity caused precocious pollen germination in *inositol polyphosphate 5-phosphatase 12* mutants [58]. Pollen deficient in the *NMNAT* gene, encoding a key enzyme in NAD biosynthesis, often precociously germinates in the locule under high-humidity conditions [62]. All these findings indicate that hydration/humidity is essential for pollen germination and that various components affecting pollen adhesion and hydration in response to environmental humidity are also required. An interesting phenotype, precocious pollen germination within *fla14* (Fig. 6) and *agp6 agp11* anthers [54], indicated that accumulation of AGPs during pollen maturation is important to repress pollen tube germination under high-humidity conditions.

In male–female cross-talk during pollen germination, the pollen grain has to hydrate, and the stigma serves as a source of water and other factors. Concurrently, the pollen coat must provide lipids and proteins required for this crucial hydration step [54]. Microscopic observations of *fla14* showed no defects in female organ function (Fig. 7). Moreover, mutation of *FLA14* did not seem to produce any conspicuous effect on the deposition of the pollen coat material according to the TEM observations of mutant pollen grains (Fig. 9). All these results indicated that pollen contact with the stigma may not be compromised in the mutant plants. It has been suggested that AGPs are involved in the control of pollen germination possibly by a relatively simple process of modulating access to water for hydration or by interfering with some kind of signaling pathway [23]. Therefore, the early germinating pollen tubes inside the *fla14* anther may be attributed to the early uptake of water or other factors.

It has been reported that during pollen germination, pollen is first activated by the uptake of water and calcium ions (Ca^2+^) into the pollen grain, and then a cytoplasmic gradient of Ca^2+^ forms beneath the site of germination [63]. In accordance with this, the expression levels of calcium- and signaling-related genes were found to be altered in double null mutant *agp6 agp11* pollen tubes, indicating the putative involvement of AGPs in signaling cascades through calmodulin [64]. Further investigation confirmed that AGPs can bind Ca^2+^, which is released in specific stages of pollen germination and tube elongation, to form an AGP-Ca^2+^ oscillator [65, 66]. Moreover, the properties of AGPs combined with Hechtian strand-mediated adhesion offer a solution based on a Hechtian oscillator that dissociates periplasmic AGP-Ca^2+^, resulting in Ca^2+^ influx [67]. The Hechtian hypothesis proposes that AGP functions as a Ca^2+^ capacitor and pollen tube guide that regulates pollen tip growth [67]. Subcellular localization of FLA14 showed that FLA14 was distributed on the plasma membrane and in Hechtian strands (Fig. 2). This result is consistent with the presence of a putative GPI anchor signal sequence predicted from the gene (Additional file 2: Fig. S2). Hechtian strands in the adhesion zones connect the cell wall to the plasma membrane, mechanically transduce cell wall stress signals to receptors located in the plasma membrane and are suggested to be implicated in signal transduction, cell–cell communication events and sensing cell wall integrity [68, 69]. Several members of the AGP family, such as LeAGP1 in tomato and FLA4, AtAGP17 and AtAGP18 in Arabidopsis, have been found to be distributed on the plasma membrane and in Hechtian strands and are proposed to be a kind of medium connecting the plasma membrane and cell wall [14, 68, 70–73]. The plasma membrane and Hechtian strand localization of FLA14 was implied to mediate its function in cell adhesion and signaling during pollen germination [36]. It has been hypothesized that external dynamic storage by a periplasmic AGP flux capacitor may be associated with the adaptation of the cell to changing conditions and its response to stress factors [27, 66, 67]. Based on this hypothesis, we speculate that interference of the plasma membrane and Hechtian strand-localized FLA14 might disturb the sensing of external signals and disrupt periplasmic AGP-Ca^2+^ dissociation, as well as destabilize networks in pollen grains and pollen tubes, causing failure in the pollen tube response to high humidity and prevention of premature pollen germination inside the anthers. It would be interesting to investigate whether the *fla14* mutant may have any defect in the expression of calcium- and signaling-related genes that are critical for pollen germination and tube growth.

## Conclusions

In this study, we characterized an Arabidopsis FLA member, FLA14, a pollen-specific protein localized at the plasma membrane and in Hechtian strands. In addition, we demonstrated a role for *FLA14* in male gametophyte development and pollen germination inside the anthers under high relative humidity. These findings provide novel insights into the implications of AGPs in plant reproductive development.

## Methods

### Plant materials and growth conditions

An ecotype of Arabidopsis with a Columbia-0 background, as well as T-DNA mutant and transgenic plants, were cultivated in potting mix in a 22 ± 2 °C growth cabinet with 50% relative humidity and a 16-h light and 8-h dark cycle.

### Phylogenetic analysis and expression analysis of FLA genes in Arabidopsis

The 21 FLA protein sequences were obtained from the TAIR (https://www.arabidopsis.org/) database. Multiple alignments of amino acid sequences were performed using the Clustal X (version 1.83) program. A phylogenetic tree was constructed in MEGA X software with the neighbor-joining algorithm method. The bootstrap value was 1000. The generated.nwk file was then used as the input to construct the final phylogenetic tree using the OmicStudio tools at https://www.omicstudio.cn/tool/.

The gene expression data for all 21 FLAs were obtained from the AtGenExpress Consortium (Arabidopsis eFP Browser, available at http://www.bar.utoronto.ca/) in ATH1 and Klepikova data sources and previously published articles [37, 38, 74, 75]. A hierarchical clustering heatmap was produced to visualize the gene expression level using the OmicStudio tools at https://www.omicstudio.cn/tool/.

### Bioinformatic analysis

The amino acid composition of FLA14 was computed using the ProtParam tool (http://web.expasy.org/protparam/). Pairwise sequences were compared by the Align tool (http://www.ebi.ac.uk/). The SignalP 4.0 Server (http://www.cbs.dtu.dk/services/SignalP-4.0/), the TMHMM Server v. 2.0 (http://www.cbs.dtu.dk/services/TMHMM/), and SMART (http://smart.embl-heidelberg.de) were used to determine the predicted length of the N-terminal signal sequence of FLA14. Original prediction for the GPI modification site was performed using GPI-SOM (http://gpi.unibe.ch) and BIG-PI Plant Predictor (http://mendel.imp.ac.at/gpi/plant_server.html). The *cis*-acting elements in the *FLA14* promoter sequence were identified using the PlantCare (http://bioinformatics.psb.ugent.be/webtools/plantcare/html). WoLF PSORT (http://wolfpsort.org/) was used to determine protein subcellular localization. Other information on *FLA14* was obtained from NCBI (http://www.ncbi.nlm.nih.gov).

### RNA extraction, cDNA synthesis and quantitative real-time PCR

Total RNA was extracted using Trizol® reagent (Invitrogen, http://www.lifetechnologies.com) and transcribed into cDNA with a PrimeScript® RT Reagent Kit with gDNA Eraser (TaKaRa, http://www.takarabiomed.com.cn). Then, the cDNA was used as the template for PCR analysis with specific primers (Additional file 4: Table S1). *Tubulin beta-4* (*Tub4*) was used as the normalization control [52]. Quantitative real-time PCR was performed in a mixture containing SYBR® Premix Ex TaqTM (TliRNaseH Plus) (TaKaRa, http://www.takarabiomed.com.cn) on a BioRad CFX96 Real-time RT-PCR Detection System (Bio-Rad, http://www.bio-rad.com). Three technical repeats and three biological replicates were performed. The values represent the means ± SD (standard deviation) of three biological replicates. The relative expression levels were calculated by the 2^−△△Ct^ method [76].

### GUS staining analysis and GFP fluorescence microscopy

For analysis of *FLA14* promoter activity, we first constructed a *pBI101-eGFP-GUS* empty vector as follows: the *enhanced green fluorescent protein* (*eGFP*) gene coding ORF fragment with the modified stop codon was PCR-amplified using specific primers and cloned into plasmid pBI101 at the *Sma*I recognition site upstream of the *β-glucuronidase* (*GUS*) gene. The *proFLA14::eGFP-GUS* construct was generated by amplifying the *FLA14* promoter sequence 2074 bp upstream from the first ATG of the *FLA14* coding ORF fragment using gene-specific primers. The fragment was inserted upstream of the *eGFP* gene at the *BamH*I recognition site in the pBI101 plasmid, which contained the *NPTII* kanamycin resistance gene. All primers are listed in Additional file 4: Table S1. Homozygous transgenic plants were used for GUS staining with 5-bromo-4-chloro-3-indolyl-*β*-glucuronic acid and GFP observation. Images of GUS-stained and GFP-expressing tissues were collected using a fluorescence microscope (ECLIPSE 90i; Nikon).

### Subcellular localization

To observe FLA14 subcellular distribution, we amplified the *FLA14* signal peptide sequence and the rest of the *FLA14* ORF fragment. Then, these two sequences were subcloned into the 3’- and 5’-termini of *GFP* in the *pFGC-eGFP* vector under the control of the constitutive CaMV 35S promoter to avoid disturbing the potential function of the signal peptide, resulting in the modified vector *pFGC-NS*^*FLA14*^*-eGFP-FLA14*. All primers are listed in Additional file 4: Table S1. The modified vector was then transiently transformed into onion epidermal cells by particle bombardment [77]. The onion epidermal layer was peeled 24 h after introduction and plasmolyzed in 0.3 g·mL^–1^ sucrose for 3 min. The expression of eGFP-fusion protein was analyzed with a fluorescence microscope (ECLIPSE 90i; Nikon) and an LSM780 confocal microscope (ZEISS, Germany).

### Mutant verification

Mutant seeds of two lines, CS1014037 and SALK_123695, were obtained from the Arabidopsis Biological Resource Center (http://www.biosci.ohio-state.edu/pcmb/Facilities/abrc/abrchome.htm). Genomic DNA was isolated from fresh young leaves using the cetyl trimethyl ammonium bromide extraction method [78]. Homozygous lines were screened by PCR with *FLA14*-specific primers (Additional file 4: Table S1) and T-DNA primer (5’-TTCTCATCTAAGCCCCCATTTGG-3’). The PCR products were confirmed by sequencing. Total RNA from homozygous mutants was extracted, and quantitative real-time PCR was performed to analyze the expression of *FLA14* according to the method described above with three specific primer pairs (Additional file 4: Table S1).

### Construction of *FLA14*-overexpressing recombinant plasmid and plant transformation

For the overexpression construct, the ORF of *FLA14* was amplified and subcloned into the pBI121 vector under the control of the constitutive CaMV 35S promoter at the *Xba*I and *Sma*I recognition sites, which flanked the *NPTII* kanamycin resistance gene. After sequencing, the recombinant construct was transformed into Arabidopsis using the floral dip method [79]. T_2_ generation plants were used for further experiments after verification. All primers used are listed in Additional file 4: Table S1.

### Light microscopy

A week after the first anthesis, florets were collected and observed with a Leica MZ16FA stereoscopic microscope (http://www.leica.com/). Siliques were fixed with 1:9 acetic acid/ethanol overnight at 4 °C, transferred to 90% ethanol for 1 h at room temperature, and left in 70% ethanol overnight at 4 °C [80]. Ethanol-fixed siliques were visualized under a Leica MZ16FA stereoscopic microscope (http://www.leica.com/).

For cytological analysis, 4’,6-diamidino-1-phenylindole (DAPI) solution was used to detect the nuclei of pollen grains [81]. Pollen viability was observed by staining with Alexander stain [82]. To observe the callose wall, pollen grains were labeled with 0.1% (w/v) aniline blue in 0.1 M K_2_HPO_4_-KOH buffer (pH 11) according to the manufacturer’s instructions (Roche, Germany). To trace the deposition of cellulose-like polysaccharides in the pollen wall, anthers and pollen grains were fixed in 5% (v/v) formalin and 5% (v/v) glacial acetic acid in ethanol overnight and then stained in calcofluor white for 5 min [83]. The stained specimens were observed with a Leica DMLB fluorescence microscope (http://www.leica.com/).

For semithin section analyses, floral buds were fixed and prepared as described by Lin et al. [50]. After embedding in spurr resin, semithin sections (1 μm) were obtained under an LKB 11800 PYRAMITOME ultramicrotome (Stockholm, Sweden), placed on glass slides, stained with 0.5% toluidine blue, and photographed by a Leica DMLB fluorescence microscope (http://www.leica.com/).

### Electron microscopy

For scanning electron microscopy (SEM), anthers at the anthesis stage and mature pollen grains were spread, coated with gold–palladium in an Eiko Model ion coater (Ibaraki, Japan) for 5 min, and then observed in a Hitachi Model TM-1000 SEM (Tokyo, Japan). For transmission electron microscopy (TEM), anthers were fixed and embedded as described above. Ultrathin sections (70 nm) were obtained using an ultramicrotome (Reichert-Jung Ultracut E, Vienna, Austria) and stained with uranyl acetate and alkaline lead citrate for 15 min. Images were recorded with a Hitachi Model H-7650 TEM (Tokyo, Japan).

### Pollen germination assays

For pollen germination assays, three-week-old Arabidopsis plantlets were grown under controlled conditions at 50% relative humidity, while another subset of plants was transferred to a growth cabinet set at 85% relative humidity. The anthers were collected 3 days after high humidity treatment and subjected to microscopy observation as described above.

### Self and reciprocal crosses

For self and reciprocal crosses, florets were pre-emasculated and pollinated 2 days before anthesis. Pistils were excised 4, 12, and 24 h after pollination, fixed in Carnoy’s solution (ethanol:alcohol acetic = 3:1) for 2 h, washed three times with water, softened with 8 M NaOH overnight, washed three additional times with water, and incubated for 3 h in 0.1% (w/v) aniline blue in 0.1 M K_2_HPO_4_-KOH buffer (pH 11). Three replicates were performed for each crossing. The specimens were observed with a Leica MZ16FA stereoscopic microscope (http://www.leica.com/).

## Supplementary Information

**Additional file 1:**

**Additional file 2:**

**Additional file 3:**

**Additional file 4:**

**Additional file 5:**

## Data Availability

The datasets used and/or analysed during the current study available from the corresponding author on reasonable request.

## Abbreviations

AGP: Arabinogalactan protein
Ca^2^^+^: Calcium ions
DAPI: 4’,6-Diamidino-1-phenylindole
eGFP: Enhanced green fluorescent protein
FAS: Fasciclin
FLA: Fasciclin-like AGP
GPI: Glycosyl phosphatidylinositol
GUS: β-Glucoronidase
OE: Over-expression
ORF: Open reading frame
SEM: Scanning electron microscopy
SD: Standard deviation
TEM: Transmission electron microscopy
*Tub4*: *Tubulin beta-4*
WT: Wild-type
