# Supplementary material for: FLA14 is required for pollen development and preventing premature pollen germination under high humidity in Arabidopsis
Source: BMC Plant Biol. 2021 Jun 3;21:254. doi: 10.1186/s12870-021-03038-x (PMC8173729; doi:10.1186/s12870-021-03038-x)
Supplement: Supplementary file 1 — Additional file 1: [file 12870_2021_3038_MOESM1_ESM.pdf]

Supplementary Material File

FLA14 is required for pollen development and preventing premature pollen germination under high humidity in Arabidopsis

Yingjing Miao[a, b], Jiashu Cao[b], Li Huang[b], Youjian Yu[c], Sue Lin[a, d*]

[a] *Institute of Life Sciences, College of Life and Environmental Science, Wenzhou University, Wenzhou 325000, China*

[b] *Laboratory of Cell & Molecular Biology, Institute of Vegetable Science, Zhejiang University, Hangzhou 310058, China*

[c] *College of Agriculture and Food Science, Zhejiang A & F University, Lin'an 311300, China*

[d] *Biomedical Collaborative Innovation Center of Zhejiang Province, Wenzhou 325000, China*

*Correspondence author at: Institute of Life Sciences, College of Life and Environmental Science, Wenzhou University, Wenzhou 325000, China.

Telephone: +8657786591693; e-mail address: iamkari@163.com (Sue Lin).

Additional file 1:

Figure S1 Phylogenetic relationships and expression analysis of 21 FLAs in Arabidopsis.

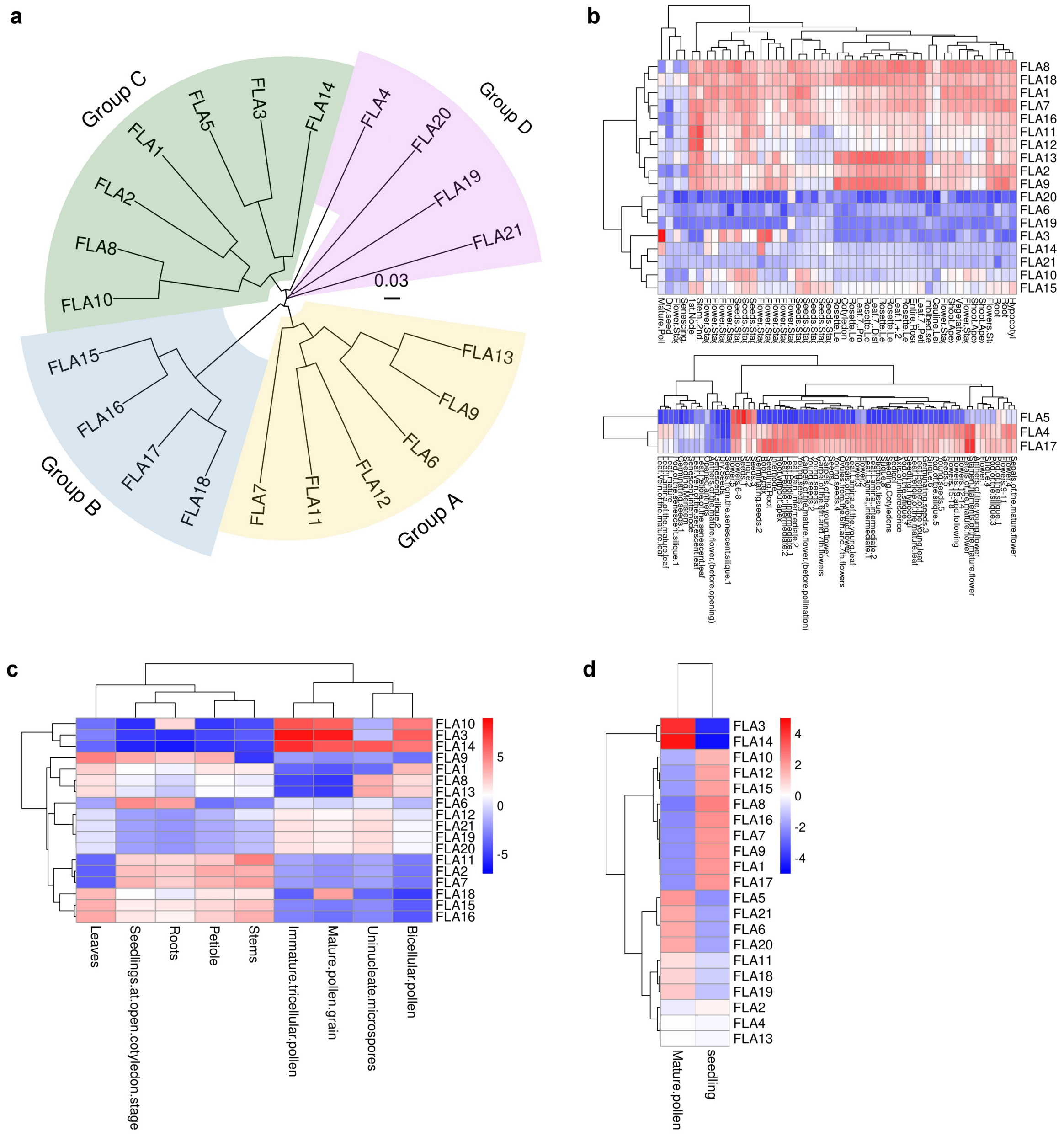

Fig. S1 Phylogenetic relationships and expression analysis of 21 FLAs in Arabidopsis. a Phylogenetic relationships of 21 FLAs. The phylogenetic tree is based on the multiple sequence alignments of 21 FLAs, constructed using the neighbor-joining method with 1,000 bootstrap in the MEGA X software and then beautified with the OmicStudio tools. Four groups of FLAs are shown on different color background. **b-d** Heatmap of expression levels

of 21 FLA genes in different tissues of Arabidopsis. **b** The gene expression data for all 21 FLAs obtained from the AtGenExpress Consortium (Arabidopsis eFP Browser). **c** Transcriptome data for 18 FLA genes identified using Affymetrix ATH1 genome arrays in Arabidopsis by Honys and Twell (2004, Genome Biology, 5, R85). **d** Comparison of high-throughput sequencing data for 21 FLA genes in Arabidopsis mature pollen grains and seedlings by Loraine et al. (2013, Plant Physiology, 162, 1092-1109).
