# Supplementary material for: FLA14 is required for pollen development and preventing premature pollen germination under high humidity in Arabidopsis
Source: BMC Plant Biol. 2021 Jun 3;21:254. doi: 10.1186/s12870-021-03038-x (PMC8173729; doi:10.1186/s12870-021-03038-x)
Supplement: Supplementary file 2 — Additional file 2: [file 12870_2021_3038_MOESM2_ESM.pdf]

Additional file 2:

Figure S2 Analysis of Arabidopsis FLA14 protein sequence.

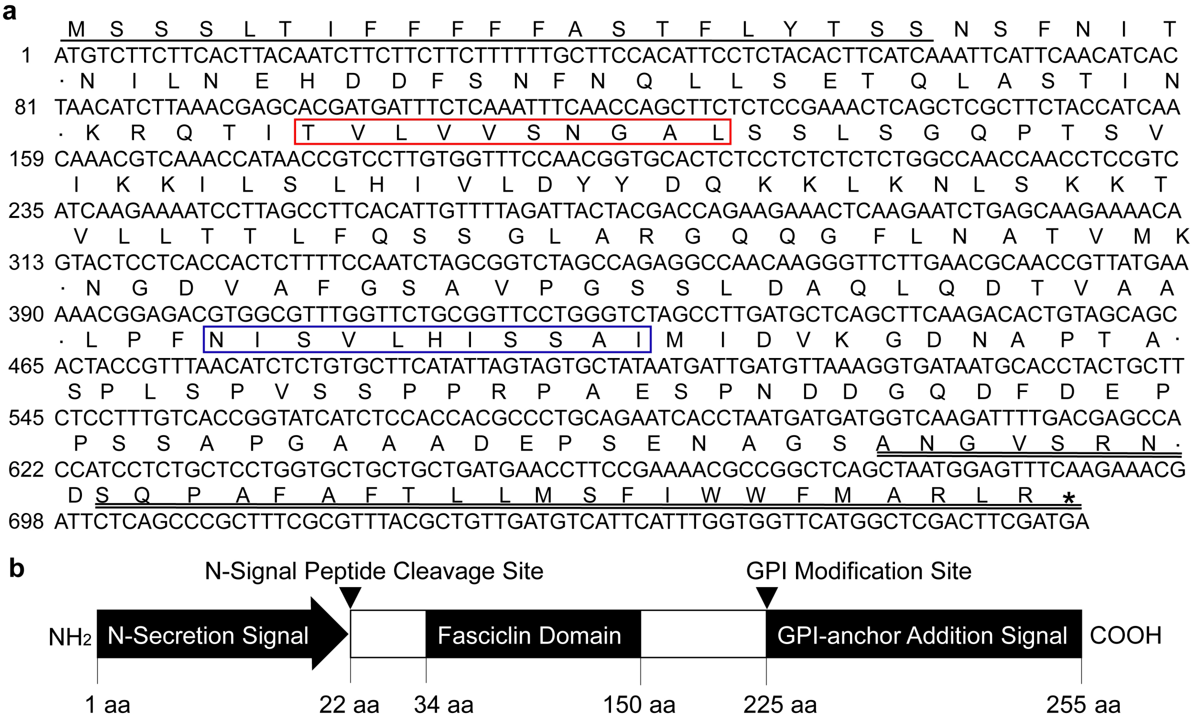

Fig. S2 Analysis of Arabidopsis FLA14 protein sequence. a The protein sequence of FLA14 deduced from the DNA sequence. The N-terminal secretion signal (underlined) and glycosyl phosphatidylinositol (GPI) anchor signal (double-underlined) are marked. Red and blue boxes indicate the conserved regions H1 and H2 of fasciclin domain, respectively. Asterisk indicates termination codon. **b** Schematic representation of FLA14 protein structure deduced from the DNA sequence.
