# Supplementary material for: FLA14 is required for pollen development and preventing premature pollen germination under high humidity in Arabidopsis
Source: BMC Plant Biol. 2021 Jun 3;21:254. doi: 10.1186/s12870-021-03038-x (PMC8173729; doi:10.1186/s12870-021-03038-x)
Supplement: Supplementary file 3 — Additional file 3: [file 12870_2021_3038_MOESM3_ESM.pdf]

Additional file 3:

Figure S3 Confirmation of Arabidopsis *FLA14* mutant and overexpression (OE) transgenic plants by genomic DNA PCR.

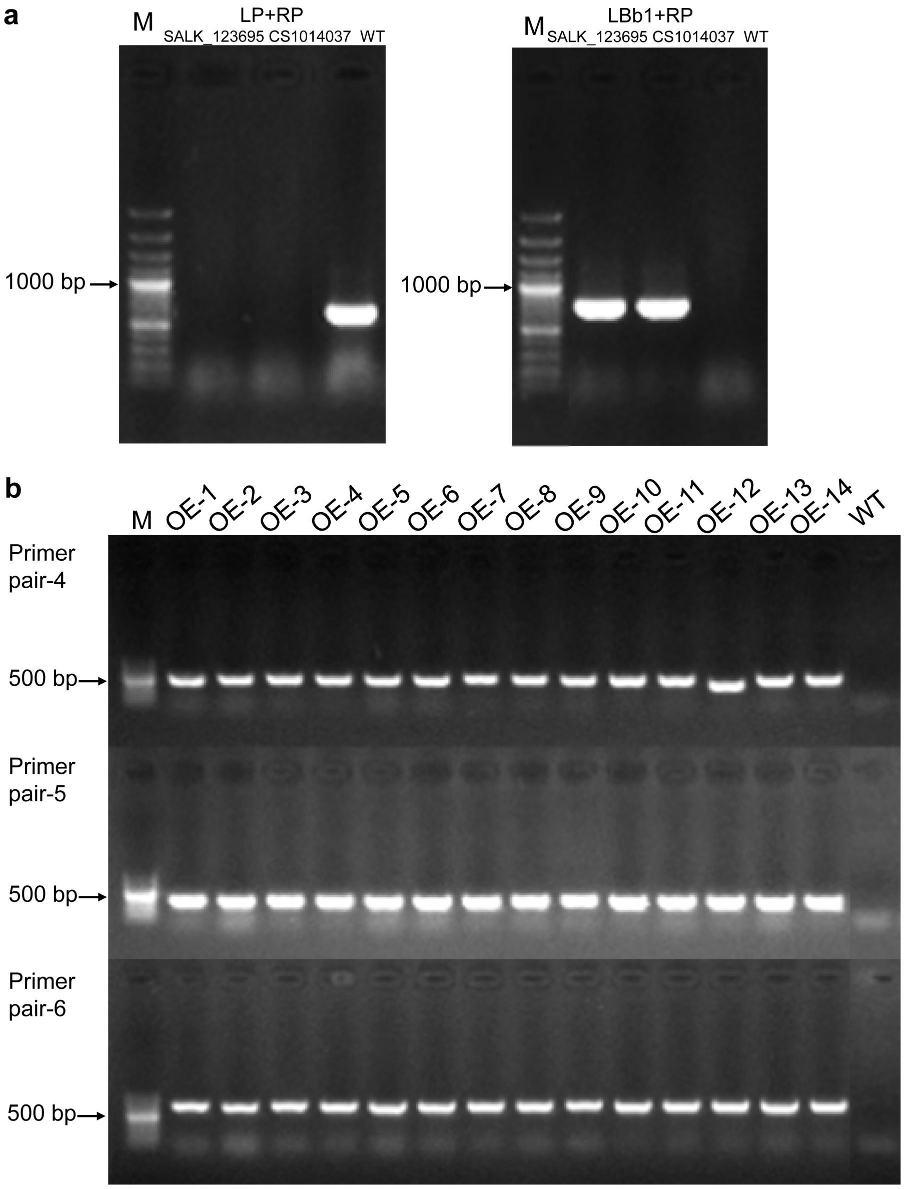

Fig. S3 Confirmation of Arabidopsis *FLA14* mutant and overexpression (OE) transgenic plants by genomic DNA PCR. a Genomic DNA PCR analysis of the homozygous CS1014037 and SALK_123695 mutants. **b** Genomic DNA PCR analysis of 14 OE lines. A 395-bp, a 505-bp and a 740-bp expected bands are amplified in 14 OE lines with three specific primer pairs, respectively. Lane M indicates molecular marker.
