# Supplementary material for: FLA14 is required for pollen development and preventing premature pollen germination under high humidity in Arabidopsis
Source: BMC Plant Biol. 2021 Jun 3;21:254. doi: 10.1186/s12870-021-03038-x (PMC8173729; doi:10.1186/s12870-021-03038-x)
Supplement: Supplementary file 4 — Additional file 4: [file 12870_2021_3038_MOESM4_ESM.pdf]

Additional file 4:

Table S1 List of primers for *FLA14* and other genes in Arabidopsis.

Gene	Experimental Procedures	Forward Primer	Reverse Primer
FLA14	RT-PCR and quantitative real-time PCR	5'-CACCATCCTCTGCTCCTG-3'	5'-TGAACCACCAAATGAATGAC-3'
Tubulin beta-4 (*Tub4*)	RT-PCR and quantitative real-time PCR	5'-AAGGCTTTCCTTCATTGGTACA-3'	5'-CTCTCCGGCTGTAGCATCTT-3'
FLA14 promoter	Analysis of *FLA14* promoter activity	5'-CGACTCTAGAGGATCCTTTGAATGTGATTGGTCGTA-3'	5'-ACCATCCCGGGGATCCTGTGAATCGCTTGATGAGT-3'
eGFP	Analysis of *FLA14* promoter activity	5'-CTAGAGGATCCCCGGGATGGTGAGCAAGGGCGA-3'	5'-GACTGACCTACCCGGGCCTTGTACAGCTCGTCCATG-3'
FLA14 N-signal sequence	Subcellular localization	5'-TTTACAATTAATGTCTTCTTCACTTACAATCTTC-3'	5'-TGCTCACCATTGATGAAGTGTAGAGGAATGTGG-3'

FLA14 lacking N-signal peptide	Subcellular localization	5’-GCTGTACAAGGGATCCAATTCATTCAACATCACTAACATCT-3’	5’-AGGGACTAGTCCCGGGTCATCGAAGTCGAGCCAT-3’
FLA14	Genomic DNA PCR analysis for mutant verification of SALK_123695	5’-CATCGAGTTCTATGCATTGGG-3’	5’-AGCAGAATCAAAATCAGACGG-3’
	Genomic DNA PCR analysis for mutant verification of CS1014037	5’-ACACACTCTCTTCCTCATGCC-3’	5’-TGGTTTCGTTGTTAGGGTTTG-3’
FLA14	Quantitative real-time PCR (primer pair-1) for mutant verification	5’-CACCATCCTCTGCTCCTG-3’	5’-TGAACCACCAAATGAATGAC-3’

	Quantitative real-time PCR (primer pair-2) for mutant verification	5’-CGAGCACGATGATTTCTCAA-3’	5’-CAAGGACGGTTATGGTTTG-3’
	Quantitative real-time PCR (primer pair-3) for mutant verification	5’-GTCAAACCATAACCGTCCTT-3’	5’-CTTGAGTTTCTTCTGGTCGTAG-3’
FLA14	Overexpression construct	5'-GCTCTAGAATGTCTTCTTCACTTACAATCTTC-3'	5'-TCCCCCGGGTCATCGAAGTCGAGCCAT-3'
T-DNA portion of the binary vector used in *FLA14* overexpression construct	*FLA14*-overexpressing transgenic line verification (primer pair-4)	5'-CCGACAGTGGTCCCAAAGAT-3' (pBI121 T-DNA-specific forward primer)	5'-CAAGGACGGTTATGGTTTG-3' (*FLA14*-specific reverse primer)

T-DNA portion of the binary vector used in *FLA14* overexpression construct	*FLA14*-overexpressing transgenic line verification (primer pair-5)	5'-CCGACAGTGGTCCCAAAGAT-3' (pBI121 T-DNA-specific forward primer)	5'-CTTGAGTTTCTTCTGGTCGTAG-3' (*FLA14*-specific reverse primer)
NPTII	*FLA14*-overexpressing transgenic line verification (primer pair-6)	5’-AGACAATCGGCTGCTCTGAT-3’	5’-TCATTTCGAACCCCAGAGTC-3’
