# Supplementary material for: FLA14 is required for pollen development and preventing premature pollen germination under high humidity in Arabidopsis
Source: BMC Plant Biol. 2021 Jun 3;21:254. doi: 10.1186/s12870-021-03038-x (PMC8173729; doi:10.1186/s12870-021-03038-x)
Supplement: Supplementary file 5 — Additional file 5: [file 12870_2021_3038_MOESM5_ESM.pdf]

1 **Additional file 5:**

2 **Figure S4 Self and reciprocal crosses between the *FLA14* knockout mutant (*fla14*) and**

3 **wild-type (WT) Arabidopsis plants.**

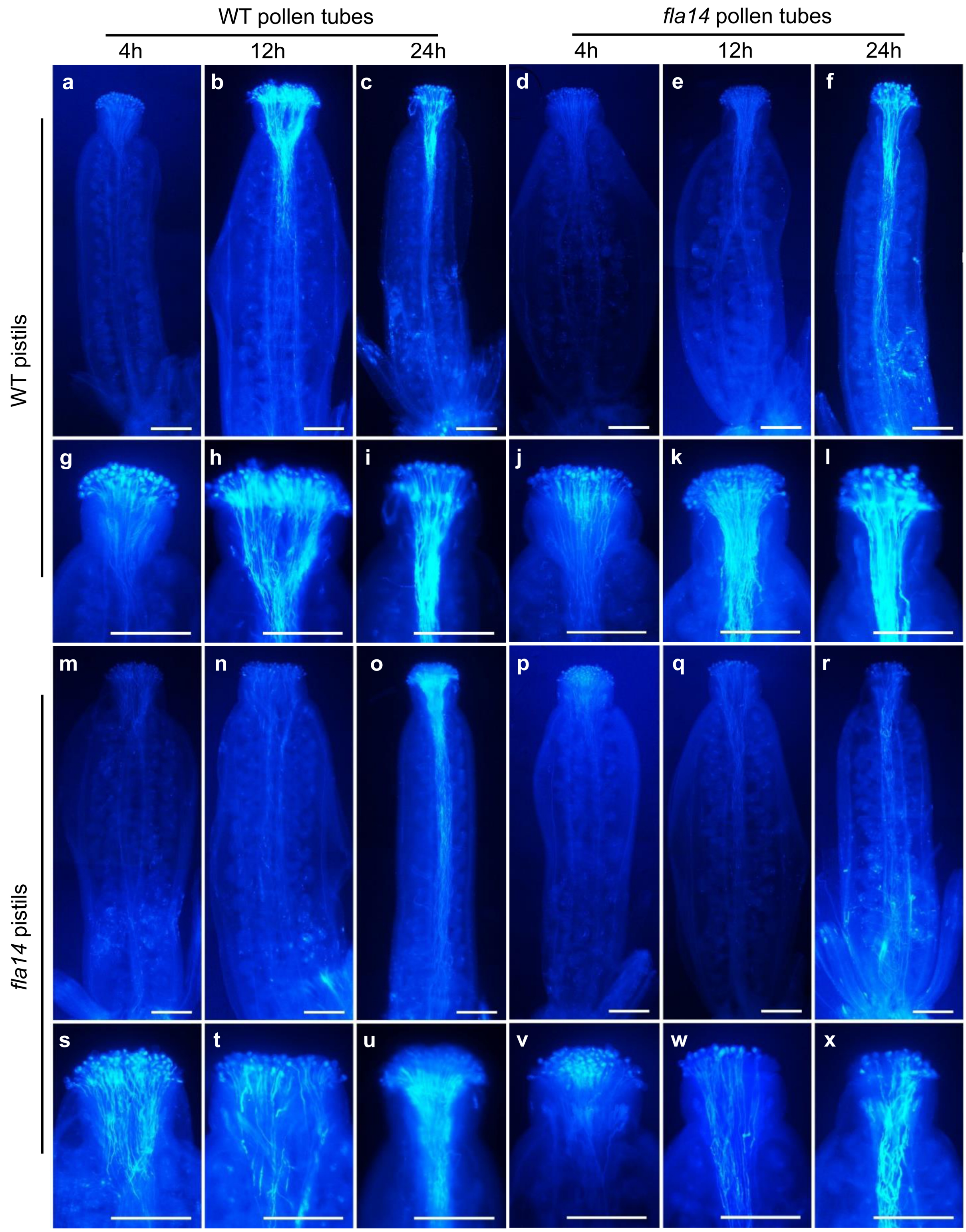

4

5 **Fig. S4 Self and reciprocal crosses between the *FLA14* knockout mutant (*fla14*) and**

wild-type (WT) Arabidopsis plants. a-c WT pollen tubes in WT pistils at 4 hours after pollination (HAP), 12 HAP, and 24 HAP, respectively. **d-f** *fla14* pollen tubes in WT pistils at 4 HAP, 12 HAP, and 24 HAP, respectively. **m-o** WT pollen tubes in *fla14* pistils at 4 HAP, 12 HAP, and 24 HAP, respectively. **p-r** *fla14* pollen tubes in *fla14* pistils at 4 HAP, 12 HAP, and 24 HAP, respectively. **g-l** and **s-x** The corresponding magnified images of (**a-f**) and (**m-r**). Scale bars = 200 μm.
